# *Jagged1/Notch2* controls kidney fibrosis via *Tfam*-mediated metabolic reprogramming

**DOI:** 10.1371/journal.pbio.2005233

**Published:** 2018-09-18

**Authors:** Shizheng Huang, Jihwan Park, Chengxiang Qiu, Ki Wung Chung, Szu-yuan Li, Yasemin Sirin, Seung Hyeok Han, Verdon Taylor, Ursula Zimber-Strobl, Katalin Susztak

**Affiliations:** 1 Renal Electrolyte and Hypertension Division, Department of Medicine, Department of Genetics, Perelman School of Medicine, University of Pennsylvania, Philadelphia, Pennsylvania, United States of America; 2 Department of Biomedicine, University of Basel, Basel, Switzerland; 3 Research Unit Gene Vectors, Helmholtz Zentrum München, German Research Center for Environment and Health, Munich, Germany; University of Cambridge Department of Pathology, United Kingdom of Great Britain and Northern Ireland

## Abstract

While Notch signaling has been proposed to play a key role in fibrosis, the direct molecular pathways targeted by Notch signaling and the precise ligand and receptor pair that are responsible for kidney disease remain poorly defined. In this study, we found that *JAG1* and *NOTCH2* showed the strongest correlation with the degree of interstitial fibrosis in a genome-wide expression analysis of a large cohort of human kidney samples. Transcript analysis of mouse kidney disease models, including folic-acid (FA)–induced nephropathy, unilateral ureteral obstruction (UUO), or apolipoprotein L1 (APOL1)-associated kidney disease, indicated that *Jag1* and *Notch2* levels were higher in all analyzed kidney fibrosis models. Mice with tubule-specific deletion of *Jag1* or *Notch2* (*Ksp*^*cre*^/*Jag1*^*flox*/*flox*^ and *Ksp*^*cre*^/*Notch2*^*flox*/*flox*^) had no kidney-specific alterations at baseline but showed protection from FA-induced kidney fibrosis. Tubule-specific genetic deletion of *Notch1* and global knockout of *Notch3* had no effect on fibrosis. In vitro chromatin immunoprecipitation experiments and genome-wide expression studies identified the mitochondrial transcription factor A (*Tfam*) as a direct Notch target. Re-expression of *Tfam* in tubule cells prevented Notch-induced metabolic and profibrotic reprogramming. Tubule–specific deletion of *Tfam* resulted in fibrosis. In summary, *Jag1* and *Notch2* play a key role in kidney fibrosis development by regulating *Tfam* expression and metabolic reprogramming.

## Author summary

Chronic kidney disease (CKD) is the ninth leading cause of death in the United States. Kidney fibrosis usually occurs at the final stage of the disease. The Notch signaling pathway is a cell–cell communication pathway, that activated by a ligand (expressed on the signal-sending cell) interacting with and inducing the breakdown of the Notch receptor on the receiving cell. We previously established that Notch signaling in renal tubular epithelial cells (RTECs) plays a key role in kidney fibrosis development. However, the precise ligand and receptor pair that contributes to kidney fibrosis remain unknown. Here, we performed a systematic analysis to define the specific molecular pathways of Notch-induced fibrosis development in RTECs. We found that *Jag1* acting together with *Notch2* leads to metabolic reprogramming of RTECs via mitochondrial transcription factor A (*Tfam*), resulting in cell proliferation and dedifferentiation and fibrosis development. Our study suggests that targeting JAG1 or NOTCH2 may have important therapeutic potential for treatment of CKD.

## Introduction

One in ten people worldwide suffers from chronic kidney disease (CKD) [1,2]. Fibrosis is the final common pathway and histological manifestation of CKD, which is characterized by the accumulation of collagen, activated myofibroblasts, inflammatory cells, epithelial cell dedifferentiation, and loss of vascular supply in the kidney [3–5].

The mechanism of fibrosis is a hotly debated issue. Renal tubule epithelial cells (RTECs) appear to play a key role in fibrosis development. Direct in vivo genetic manipulation of RTECs is both sufficient to induce or to ameliorate fibrosis development in mice [6–9]. Injury to RTECs is usually agreed to be a proximal lesion in the downstream cascade that eventually results in fibrosis [10,11]. Recent studies highlighted the reemergence of developmental pathways in fibrosis, including Notch, Wnt, and Hedgehog, in response to severe tubule injury [12–17]. While chronic activation of Notch plays a key role in development and regeneration of the kidney, sustained expression of Notch in RTECs will expand the progenitor (transit) amplifying pool but eventually block full differentiation of RTECs [18]. Epithelial dedifferentiation due to the lack of expression of key functional proteins will lead to functional decline (CKD). Direct targets of Notch signaling and the exact mechanism of Notch-induced RTEC dedifferentiation are not fully understood.

Notch signaling is a highly conserved cell–cell communication mechanism that regulates tissue development, homeostasis, and repair. In mammals, there are four Notch receptors (*NOTCH 1*–*4*) and five canonical ligands, Jagged (*JAG1*, *2*) and Delta-like ligand (*DLL1*, *3*, *4*). Upon ligand binding, the Notch intracellular domain (NICD) travels to the nucleus, binds to RBPJ (recombination signal binding protein for immunoglobulin kappa J region), and mediates gene transcription [19]. While Notch signaling plays an important role in a multitude of disease development, it also has an important homeostatic function [17]. Systemic pharmacological targeting of NOTCH (mostly via gamma secretase inhibition) has been developed and is being tested in clinical trials for the treatment of various cancer types [20]. Most of these drugs have been associated with significant toxicities, mostly gastrointestinal effects, that likely limit their use for chronic and slowly progressing conditions, for which prolonged administration is needed [20–23]. New results, on the other hand, suggest that while different Notch ligands and receptors are homologous, they seem to exert isoform-specific functions [17]. By using antibodies to specifically target Notch signaling, the Jones group found that inhibition of JAG1, acting together with NOTCH2, reduces tumor burden in a mouse model of primary liver cancer [24]. Tran and colleagues demonstrated the dominant role of DLL4–NOTCH1 signaling in T cells during graft-versus-host disease using a ligand-targeted antibody strategy [25]. Ligand-specific targeting strategies were able to dissociate disease-causing and homeostatic functions of Notch signaling; therefore, they could offer novel therapeutic strategies.

Our previous results indicated that increased and sustained tubular epithelial Notch signaling following injury plays a key role in the development of renal tubulointerstitial fibrosis (TIF) [8,26–30]. Global and systemic inhibition of Notch signaling using small molecular blockers of the gamma secretase pathway ameliorated fibrosis in the folic acid (FA), unilateral ureteral obstruction (UUO), and HIV-induced kidney fibrosis models [31–36]. Similarly, mice with genetic deletion of *Rbpj* in RTECs were protected from kidney fibrosis development [8]. As RBPJ is a common partner for all Notch receptors, these studies were unable to identify the specific Notch ligand/receptor pair responsible for the profibrotic effect of Notch signaling [17]. Furthermore, RBPJ has also been reported to have some Notch-independent activity [37,38]. Identification of specific ligands and receptors for kidney fibrosis therefore remains a critically important issue.

Here, we analyzed several mouse models and patient samples with kidney disease to understand the expression of Notch ligands and receptors. We generated mouse models with genetic deletion of individual Notch ligands and receptors, and finally, we identified direct targets of Notch signaling. We show that RTEC expression of *Jag1* acting together with *Notch2* leads to metabolic reprogramming of RTECs via mitochondrial transcription factor A (*Tfam*), resulting in epithelial dedifferentiation and fibrosis development.

## Results

### Increased *Jag1* and *Notch2* expression in RTECs of mice and patients with kidney fibrosis

**Fig 1:**
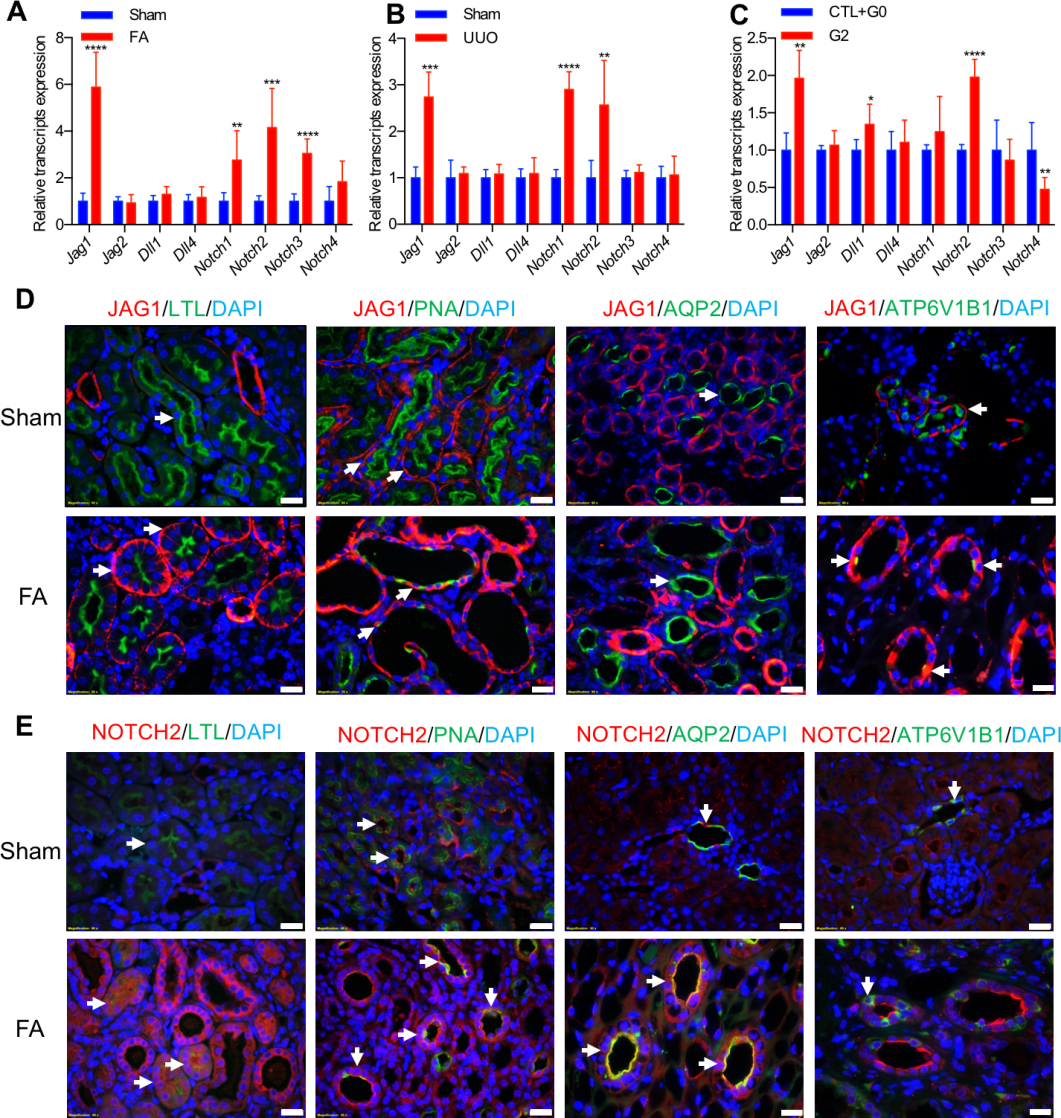
Increased *Jag1/Notch2* expression in mouse kidney fibrosis. (A–C) Relative mRNA amount of Notch ligands and receptors in whole-kidney lysates of FA-induced nephropathy group (*n* = 7, 6) (A), sham and UUO group (*n* = 5, 5) (B), and APOL1-G1/G2 mice (*n* = 4, 7) as analyzed by qRT-PCR (C). Data are represented as the mean ± SD. *P < 0.05, ** *P* < 0.01, *** *P* < 0.001, and **** *P* < 0.0001 by two-tailed Student *t* test. The underlying data of panels A–C can be found in S1 Data. (D and E) Representative images of double staining of JAG1 (D) and NOTCH2 (E) with LTL, PNA, AQP2, or ATP6V1B1 on sham or FA-treated mouse kidneys. The arrow indicates an area of interest. Scale bar: 10 μm. APOL1-G1/G2, apolipoprotein L1-G1 and G2 risk alleles; AQP2, aquaporin 2; ATP6V1B1, ATPase H+ Transporting V1 Subunit B1; DAPI, diamidino-2-phenylindole; FA, folic acid; LTL, lotus lectin; PNA, peanut agglutinin; qRT-PCR, quantitative reverse transcriptase polymerase chain reaction; UUO, unilateral ureteral obstruction.

To identify Notch ligands and receptors responsible for fibrosis development, we first evaluated the expression of Notch-pathway–associated genes in various mouse models of kidney fibrosis, including FA nephropathy [39], the UUO model [40], and podocyte-specific expression of apolipoprotein L1 (APOL1) risk variants [41]. Of the Notch ligands and receptors, expression of *Jag1* and *Notch2* was consistently and significantly higher in kidneys of the examined CKD models (Fig 1A–1C). The higher *Jag1* and *Notch2* expression was confirmed in publicly available unbiased RNA sequencing datasets as well (S1A–S1C Fig) [39–41]. Amongst the different ligands, *Jag1* transcript level was the highest in whole kidney lysates, while the level of *Dll3* was too low to detect. Of the Notch receptors, we found *Notch2* had the highest expression. We performed in situ hybridization with probes for *Jag1* and *Notch2* to validate their higher transcript level in the FA nephropathy model (S1D–S1G Fig).

Given that Notch is activated by proteolytic cleavage, we next examined JAG1 and NOTCH2 level in the kidneys of FA-induced kidney fibrosis mice by immunostaining. We confirmed the higher protein expression of JAG1 and NOTCH2 in kidneys with fibrosis (S1H and S1I Fig). Double immunostaining of JAG1 with kidney-segment–specific markers identified JAG1 mainly expressed in peanut agglutinin- (PNA, a marker for distal tubule/loop of Henle) and ATPase H+ Transporting V1 Subunit B1- (ATP6V1B1, a marker for intercalated cells of collecting duct) positive tubules, but not in lotus-lectin- (LTL, a marker for proximal tubule) and aquaporin-2 (AQP2, a marker for principal cells of collecting duct)–expressing tubule segments in healthy condition. In disease states, we observed significant higher protein level of JAG1 both on the basal and lateral membrane of LTL-, PNA-, and ATP6V1B1-positive tubule cells (Fig 1D). NOTCH2 was mainly expressed in PNA- and AQP2-positive tubule cells in the healthy condition, and it significantly increased both on the apical and lateral membrane of LTL-, PNA-, and AQP2-positive tubule segments in the FA model, suggesting that the lateral aspect is likely the site for JAG1/NOTCH2 interaction (Fig 1F).

**Fig 2:**
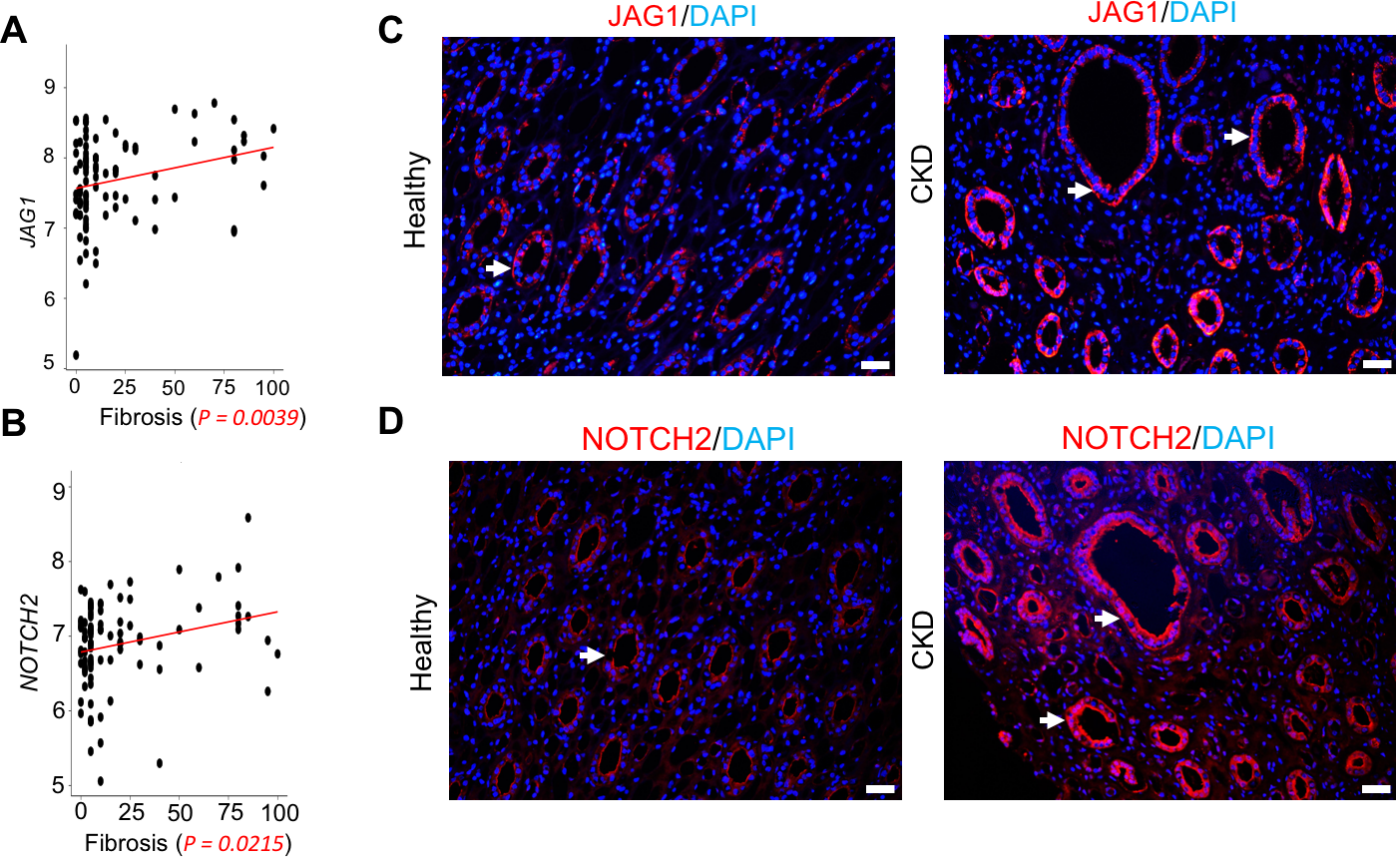
Increased *Jag1/Notch2* expression in human kidney fibrosis. (A and B) Correlation between interstitial fibrosis and *JAG1* (A) or *NOTCH2* (B) transcript level in 95 microdissected human kidney samples. The underlying data of panels A and B can be found in S1 Data. (C and D) Representative images of immunofluorescence staining with antibodies against JAG1 (C) and NOTCH2 (D) in healthy and diseased human samples. Arrows indicate positive staining in RTECs. Scale bar: 20 μm. CKD, chronic kidney disease; RTEC, renal tubular epithelial cell.

To understand the human translatability of the studied mouse models, we have analyzed gene expression changes in 95 microdissected human kidney tubule samples. The dataset included microarray samples from healthy, diabetic, and hypertensive subjects and individuals with varying degrees of fibrosis [42]. Among all tested Notch ligands, *JAG1* expression showed significant positive correlation with the degree of fibrosis (*P* = 0.0039) (Table 1; Fig 2A). We also found a positive association between the expression of *NOTCH2* with the degree of interstitial fibrosis (*P* = 0.0215) (Table 1; Fig 2B).

**Table 1:** Correlation between interstitial fibrosis and Notch signaling transcript level in 95 microdissected human kidney tubule samples.

	*β*	*P*
*JAG1*	0.0078	0.0039
*JAG2*	0.0031	0.0889
*NOTCH1*	−0.0001	0.9313
*NOTCH2*	0.0062	0.0215
*NOTCH3*	0.0029	0.2675
*NOTCH4*	0.0039	0.1085

Immunostaining studies indicated higher protein expression of JAG1 in RTECs of human TIF samples. Only a few cells were positive for JAG1 in healthy human kidney samples, but its expression was much increased in RTECs of kidneys obtained from subjects with CKD (Fig 2C). Similar results were obtained for NOTCH2 (Fig 2D). The pattern of expression in human kidneys was similar to that observed in mouse models.

In summary, *Jag1* and *Notch2* were the highest-expressed Notch ligand and receptor in whole-kidney lysates, and both their transcript and protein levels showed a consistent increase in kidney tubule cells in patient samples and in animal models of kidney fibrosis.

### TIF development is mediated by tubule epithelial expression of *Jag1* and *Notch2*

**Fig 3:**
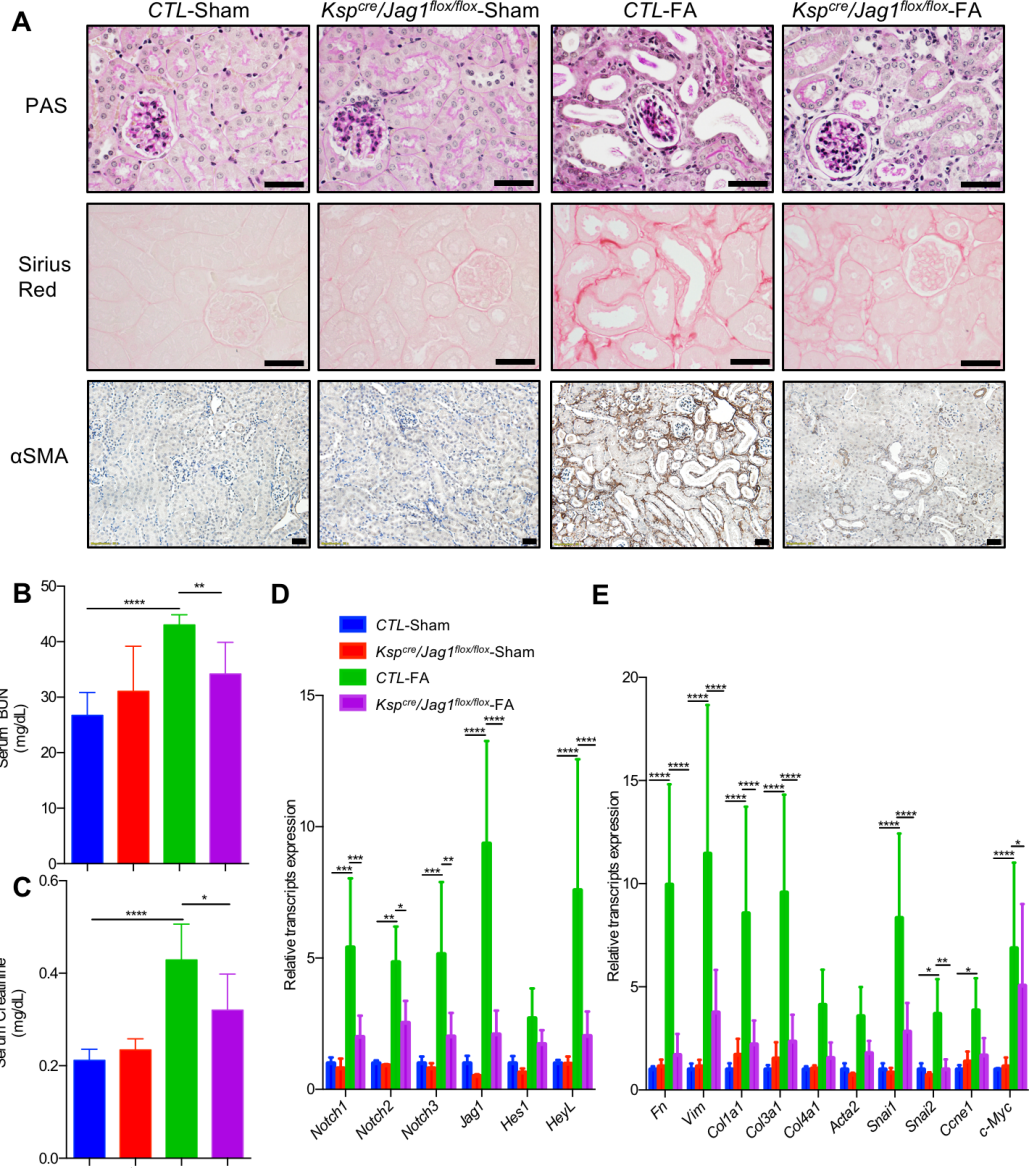
Reduced fibrosis in tubule-specific *Jag1* knockout mice. (A) Representative images of PAS-, Sirius Red-, and αSMA-stained kidney sections from CTL and *Ksp*^*cre*^/*Jag1*^*flox*/*flox*^ mice with or without FA injection. Scale bar: 20 μm. (B and C) Serum BUN and creatinine measurement of CTL and *Ksp*^*cre*^ *Jag1*^*flox*/*flox*^ mice with or without FA injection. Data are represented as mean ± SD. * *P* < 0.05, ** *P* < 0.01, and **** *P* < 0.0001 by one-way ANOVA with post hoc Tukey test (*n* = 5, 3, 9, 10). (D and E) Relative mRNA amount of Notch signaling (D), fibrosis markers, dedifferentiation, and proliferation (E) in CTL and *Ksp*^*cre*^ *Jag1*^*flox*/*flox*^ mice with or without FA injection. Data are represented as mean ± SD. * *P* < 0.05; ** *P* < 0.01, *** *P* < 0.001, and **** *P* < 0.0001 by two-way ANOVA with post hoc Tukey test (*n* = 5, 3, 9, 10). The underlying data of panels B–E can be found in S1 Data. αSMA, alpha smooth actin; BUN, blood urea nitrogen; CTL, control; FA, folic acid; PAS, Periodic acid–Schiff.

Next, we studied whether higher JAG1 expression observed in CKD renal tubule cells contributes to fibrosis development. Therefore, we crossed the *Jag1*^*flox*/*flox*^ animals with mice expressing Cre recombinase under the cadherin 16 promoter to generate animals with *Jag1* deletion in RTECs (*Ksp*^*cre*^*/Jag1*^*flox*/*flox*^) (S2A Fig) [43]. *Ksp*^*cre*^/*Jag1*^*flox*/*flox*^ mice showed lower *Jag1* expression **(**Fig 3D and S2B Fig**)** but no significant histological alterations at baseline (Fig 3A). Kidney fibrosis was induced by FA injection. We found that 7 days after FA injection, kidney histology was markedly improved in the *Ksp*^*cre*^/*Jag1*^*flox*/*flox*^ mice when compared to control (CTL) animals, as evidenced by Periodic acid–Schiff (PAS)-, Sirius Red-, and αSMA (alpha smooth muscle actin)-stained kidney sections (Fig 3A, S2C and S2D Fig). Serum blood urea nitrogen (BUN) and creatinine indicated functional improvement in tubule-specific *Jag1* knockout mice (Fig 3B and 3C). There was a marked reduction in the expression of *Notch1*–*3* and Notch target genes including *HeyL* in FA-treated *Ksp*^*cre*^/*Jag1*^*flox*/*flox*^ mice, indicating the successful reduction of Notch signaling. Epithelial dedifferentiation and proliferation are the hallmarks of fibrosis. Expression levels of *Fibronectin*, *Vimentin*, *Collagen1*, *3*, *Snai1*, *Snai2*, and *c-Myc* were lower in the FA-injected *Jag1* knockout mice when compared to CTL littermates (Fig 3D and 3E).

**Fig 4:**
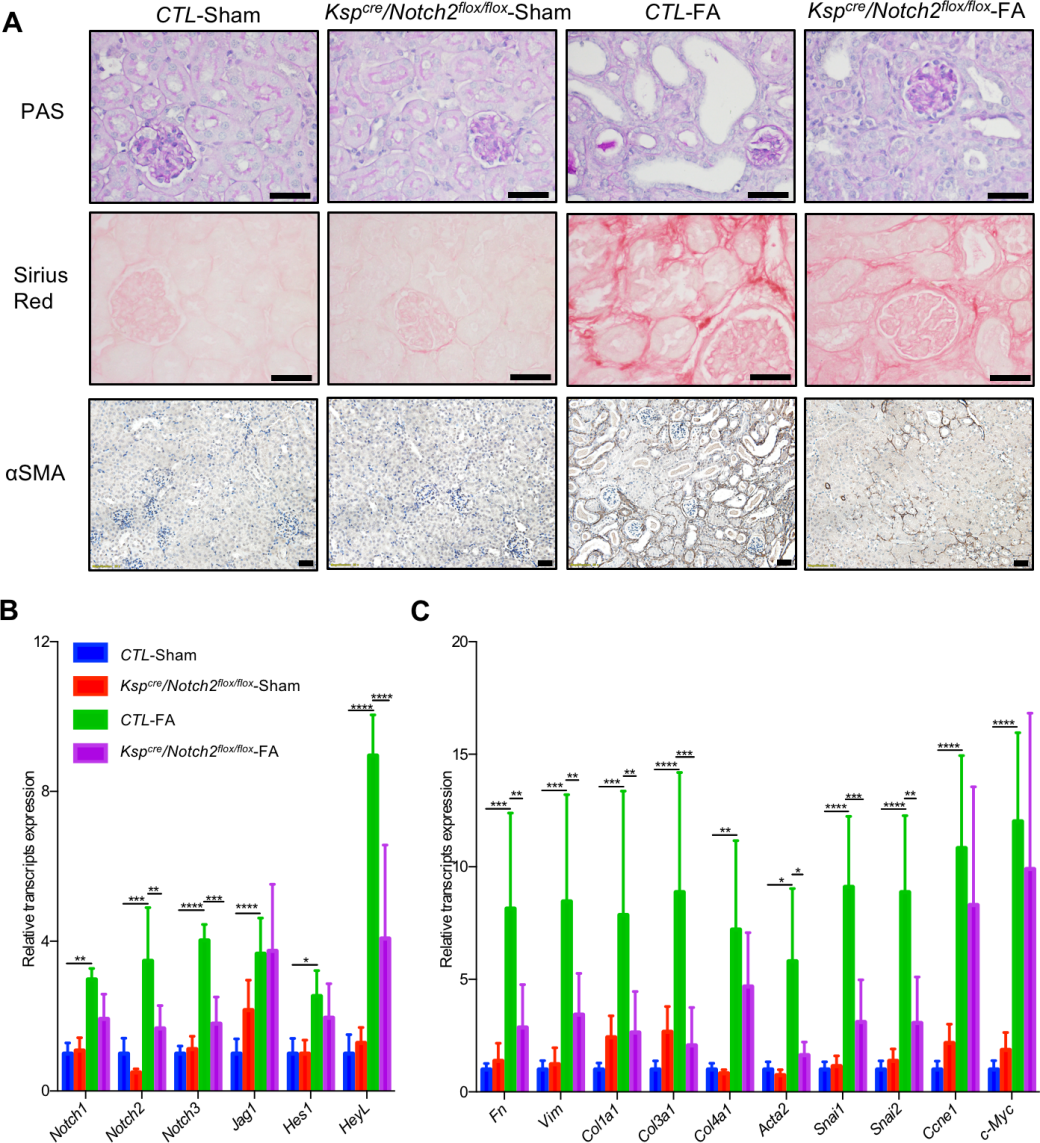
Reduced fibrosis in tubule-specific *Notch2* knockout mice. (A) Representative images of PAS-, Sirius Red-, and αSMA-stained kidney sections from CTL and *Ksp*^*cre*^/*Notch2*^*flox*/*flox*^ mice with or without FA injection. Scale bar: 20 μm. (B and C) Relative mRNA amount of Notch signaling (B), fibrosis, dedifferentiation, and proliferation markers (C) in CTL and *Ksp*^*cre*^/*Notch2*^*flox*/*flox*^ mice with or without FA injection. Data are represented as mean ± SD. * *P* < 0.05; ** *P* < 0.01, *** *P* < 0.001, and **** *P* < 0.0001 by two-way ANOVA with post hoc Tukey test (*n* = 5, 6, 5, 7). The underlying data of panels B and C can be found in S1 Data. αSMA, alpha smooth muscle actin; CTL, control; FA, folic acid; PAS, Periodic acid–Schiff.

Our unbiased analysis identified NOTCH2 as a Notch receptor with the strongest correlation with fibrosis. We next studied the role of *Notch2* in kidney fibrosis. We generated mice with RTEC-specific deletion of *Notch2* by mating the *Notch2*^*flox*/*flox*^ mice with the *Ksp*^cre^ mice (S3A and S3B Fig). Animals were born with expected Mendelian frequency, and histological analysis showed no significant difference between CTL and conditional knockout mice (Fig 4A). FA injection was used to induce kidney fibrosis. The expression of *Notch2*, *Notch3*, and *HeyL* levels were significantly lower, while *Notch1* and *Jag1* levels were unchanged in FA-treated *Ksp*^*cre*^/*Notch2*^*flox*/*flox*^ mice (Fig 4B). We found marked structural improvement when PAS-, Sirius Red-, and αSMA-stained kidney sections of FA-injected CTL and *Ksp*^*cre*^/*Notch2*^*flox*/*flox*^ were compared (Fig 4A, S3C and S3D Fig). In conjunction, levels of the profibrotic genes *Fibronectin*, *Vimentin*, *Collagen1*, *3*, *Acta2*, *Snai1*, and *Snai2* were significantly reduced in tubule-specific *Notch2* knockout animals (Fig 4C).

To understand whether this effect is specific for *Jag1* and *Notch2*, we tested the effect of genetic deletion of *Notch1* and *Notch3*. Tubule-specific *Notch1* knockout mice were generated by mating the *Ksp*^*cre*^ and *Notch1*^flox/flox^ animals. We found no significant differences in kidney fibrosis development when the *Ksp*^*cre*^/*Notch1*^flox/flox^ were compared to CTL FA-treated animals (S4 Fig). Similarly, we studied mice with global deletion of *Notch3*. We found no renal abnormalities in global *Notch3* knockout mice at baseline, and *Notch3* knockout mice showed no significant differences in FA-induced kidney fibrosis development (S5 Fig**)**. In summary, in vivo studies indicate that tubule-specific deletion of *Jag1* and *Notch2* ameliorates kidney fibrosis development in mice, while *Notch1* and *Notch3* minimally contribute to CKD.

### *Jag1*/*Notch2* in RTEC induces dedifferentiation and proliferation

**Fig 5:**
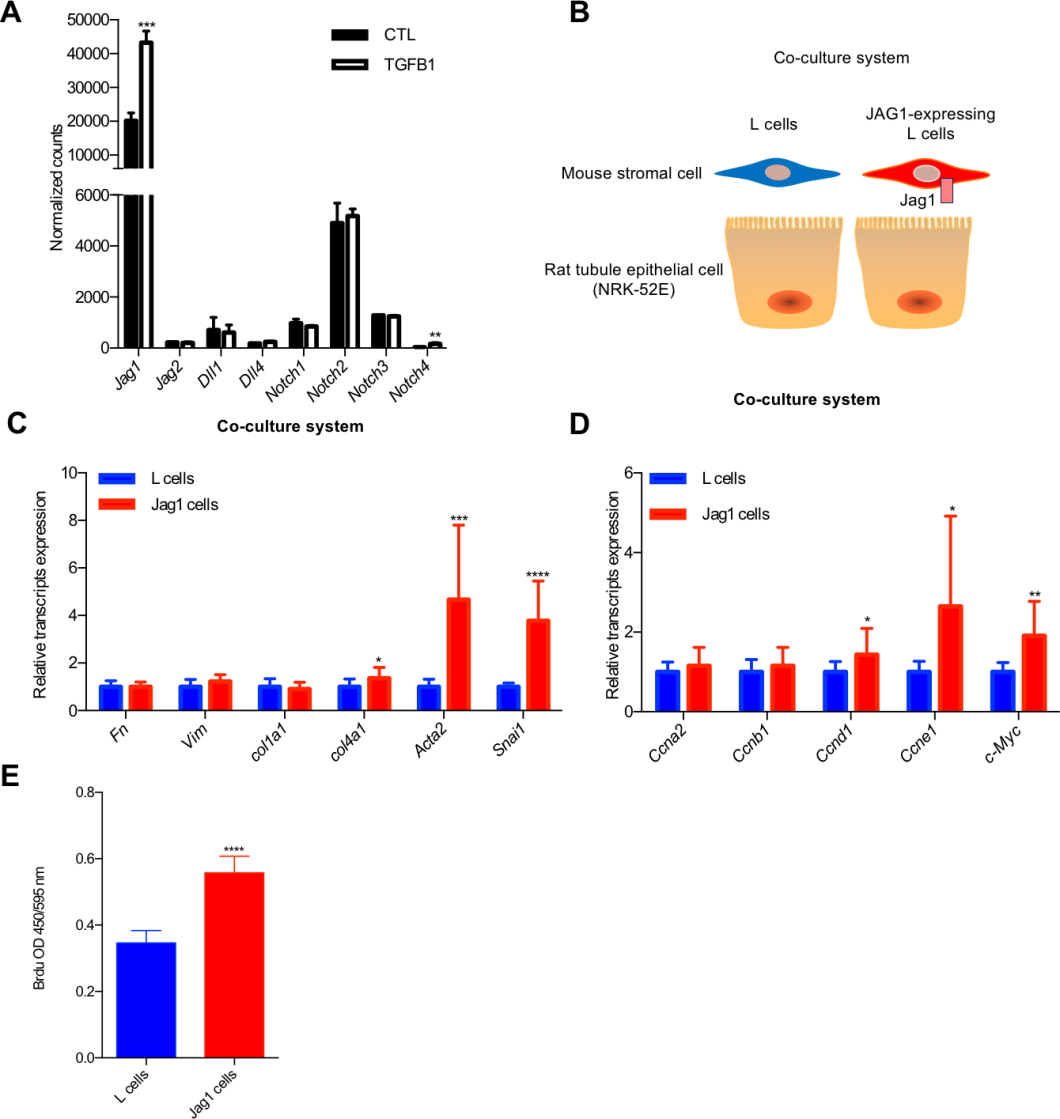
*Jag1* expression induces dedifferentiation and proliferation of RTECs. (A) Normalized counts of Notch ligands and receptor transcripts of TGF-β1–treated primary cultured mouse RTECs. Data are represented as the mean ± SD. ** *P* < 0.01 and *** *P* < 0.001 by two-tailed Student *t* test (*n* = 3 per group). (B) Experimental scheme for JAG1 co-culture system. (C and D) Co-culturing of JAG1-expressing stromal cells with rat kidney tubule (NRK-52E) cells was associated with cell dedifferentiation and proliferation. * *P* < 0.05, **P < 0.01, *** *P* < 0.001, and **** *P* < 0.0001 by two-tailed Student *t* test (*n* = 12 per group). (E) BrdU incorporation in JAG1 co-culture system. **** *P* < 0.0001 by two-tailed Student *t* test (*n* = 12 per group). The underlying data of panels A, C, D, and E can be found in S1 Data. BrdU, bromodeoxyuridine; CTL, control; RTEC, renal tubular epithelial cell.

In order to understand the downstream molecular pathways regulated by *Jag1*/*Notch2*, we turned to an in vitro system. Transforming growth factor (TGF)-β1 is one of the most powerful profibrotic cytokine and a known inducer of Notch signaling in epithelial cells by *Smad3* [8,44]. TGF-β1 treatment significantly increased *Jag1* expression in primary culture of RTECs (Fig 5A), indicating that TGF-β1 is an upstream regulator of Notch in tubule cells. Similar to the in vivo kidney expression, the expression of *Notch2* was the highest amongst the different Notch receptors in cultured RTECs.

Furthermore, to study the role of JAG1 specifically, we have set up a co-culture system by mixing RTECs with JAG1-expressing or CTL L cells (Fig 5B and S6A Fig). In this system, the ligand expressing (mouse L cells) and signal receiving cells (rat kidney epithelial cells; NRK-52E) were from different species and we used species-specific primers to discriminate gene expression levels in the signal-sending and signal-receiving cells. We found that in the JAG1 co-culture condition, Notch signaling was activated, as shown by the higher expression level of *Hes1* and *Hey1*. This activation could be blocked by the gamma secretase inhibitor dibenzazepine (DBZ) (S6B Fig).

Culturing NRK-52E with JAG1-expressing cells resulted in higher expression of markers of dedifferentiation, such as *Acta2* and *Snai1*, and enhanced proliferation, such as a higher expression of *Cyclins* and *c-Myc*, when compared to L cells (Fig 5C and 5D). Enhanced cell proliferation following JAG1 induction was confirmed by BrdU incorporation measurements (Fig 5E). The in vitro regulation of *Acta2*, *Snai1*, and *c-Myc* by JAG1/NOTCH2 recapitulated the in vivo mouse model results (Fig 3E and Fig 4C**)**. JAG1/NOTCH2 are required for epithelial dedifferentiation and proliferation both in vivo in mouse models and in vitro in tubule cells.

### *Tfam* is a direct target of Notch

**Fig 6:**
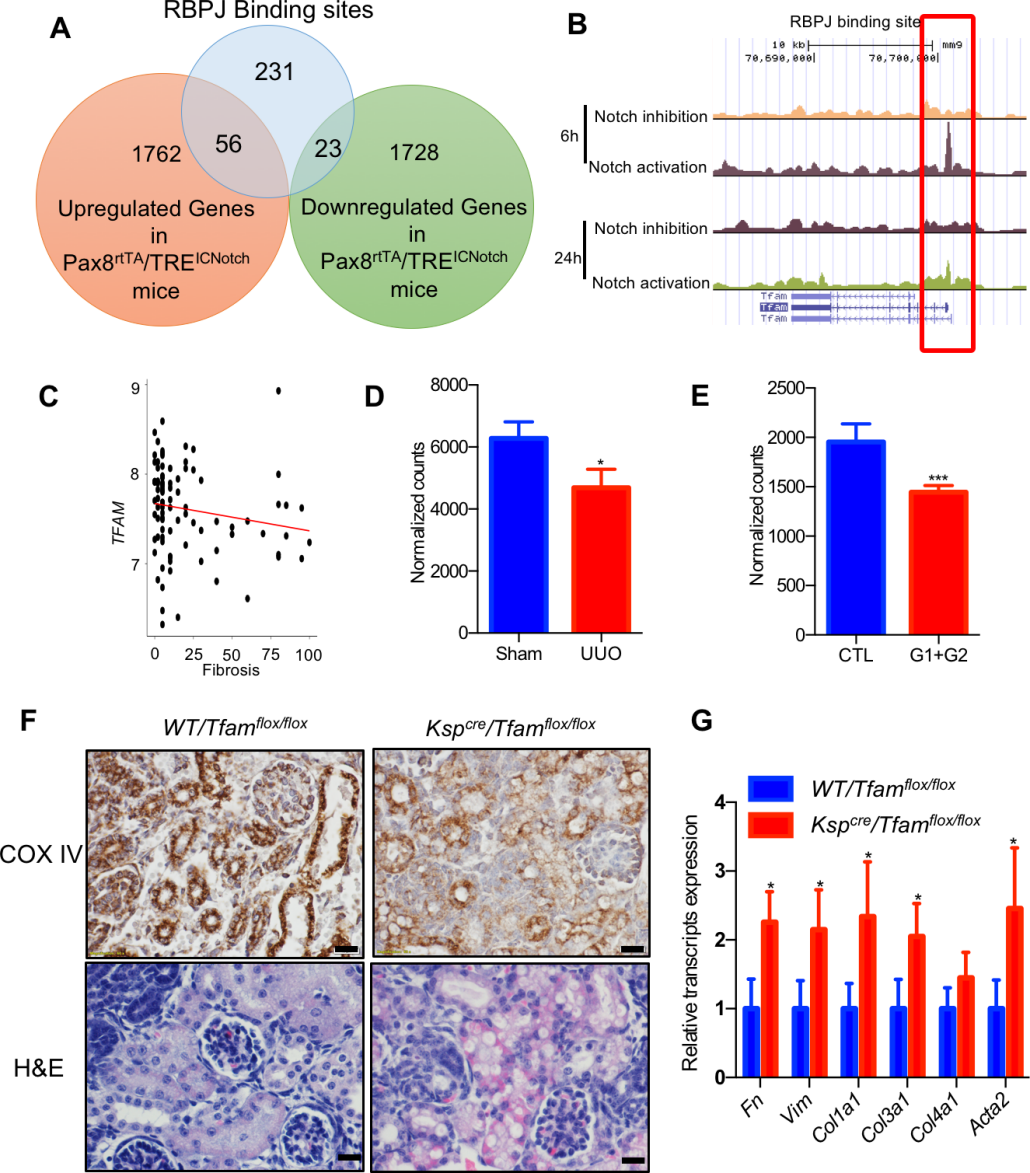
*Tfam* is the direct target of Notch and plays an important role in kidney tubules. (A) Overlap of RBPJ ChIP-seq binding peaks with differentially expressed genes in kidneys of *Pax8*^*rtTA*^/*TRE*^*ICNotch1*^ mice. (B) The mouse *Tfam* locus and RBPJ ChIP-Seq from 6 h and 24 h Notch activation and inhibition. (C) Correlation between interstitial fibrosis and *TFAM* transcript level in 95 microdissected human kidney samples. (D and E) Normalized counts of *Tfam* by RNA sequencing in whole-kidney lysates of sham and UUO group (*n* = 4, 3) (C), and APOL1-G1/G2 mice (*n* = 6, 4) (D). Data are represented as the mean ± SD. * *P* < 0.05 and *** *P* < 0.001 by two-tailed Student *t* test. (F) COX IV and H&E staining from kidneys of *Ksp*^*cre*^/*Tfam*^*flox*/*flox*^ mice and CTL littermates. Scale bar: 10 μm. (G) Relative mRNA amount of fibrosis markers in CTL and *Ksp*^*cre*^/*Tfam*^*flox*/*flox*^ mice. Data are represented as mean ± SD. * *P* < 0.05 by two-tailed Student *t* test (*n* = 4, 3). The underlying data of panels C, D, E, and G can be found in S1 Data. COX IV, cytochrome c oxidase subunit IV; CTL, control; H&E, Hemotoxylin and Eosin; RBPJ, recombination signal binding protein for immunoglobulin kappa J region; Tfam, mitochondrial transcription factor A; UUO, unilateral ureteral obstruction; WT, wild type.

To understand the direct molecular targets of Notch signaling, we have examined RBPJ chromatin immunoprecipitation sequencing datasets (ChIP-seq) [45]. To filter out functionally important binding sites that are also associated with gene expression changes in vivo, we correlated the ChIP-seq results with transcriptome changes observed in kidneys of tubule-specific NICD transgenic mice (*Pax8*^*rtTA*^/*TRE*^*ICNotch1*^) [8]. This combined analysis identified 79 RBPJ-binding sites that were associated with gene expression changed following in vivo NOTCH expression in tubule cells (56 RBPJ-binding sites with higher gene expression level and 23 RBPJ-binding sites with lower gene expression level) (Fig 6A; S1 and S2 Tables). Gene ontology classification of genes with direct RBPJ binding and RTEC gene expression changes indicated enrichment for metabolic pathways (i.e. *Rxra*, *Tfam*, *Acot12*, *Ndufa10*, etc.) (Table 2).

**Table 2:** Gene ontology analysis of overlap RBPJ-binding peaks identified in Fig 6A.

	KEGG pathway	Gene	Chromosome	Peak start	Peak end	RBPJ binding
Up-regulated genes in *Pax8*^*rtTA*^/*TRE*^*ICNotch1*^ mice	Notch signaling	*Hes1*	chr16	30063927	30064527	Inducible
			chr16	30065039	30065639	Inducible
			chr16	30066154	30066754	Inducible
		*Hey1*	chr3	8717326	8717926	Inducible
		*Heyl*	chr4	122904856	122905456	Inducible
			chr4	122905468	122906068	Inducible
			chr4	122916604	122917204	Inducible
		*Jag1*	chr2	136875009	136875609	Inducible
		*Notch1*	chr2	26349755	26350355	Inducible
	TGF-β signaling	*Bmpr1a*	chr14	35260679	35261279	Inducible
		*Smad3*	chr9	63550165	63550765	Inducible
		*Tgfb1*	chr7	26496547	26497147	Inducible
		*Tgfbr1*	chr4	47306423	47307023	Inducible
Down-regulated genes in *Pax8*^*rtTA*^/*TRE*^*ICNotch1*^ mice	Metabolic pathways	*Acot12*	chr13	91903860	91904460	Inducible
		*Atp6v1c1*	chr15	38655430	38656030	Inducible
		*Hgd*	chr16	37615579	37616179	Inducible
		*Maoa*	chrX	16281500	16282100	Inducible
		*Ndufa10*	chr1	94344895	94345495	Inducible
		*Qdpr*	chr5	45519151	45519751	Inducible
		*Rxra*	chr2	27595697	27596297	Inducible
		*Tfam*	chr10	70700475	70701075	Inducible

**Abbreviations:** KEGG, Kyoto Encyclopedia of Genes and Genomes; RBPJ, recombination signal binding protein for immunoglobulin kappa J region; TGF-β, transforming growth factor-β.

Epidermal growth factor (EGF, encoded by *Egf*) was one of the top differentially expressed genes in *Pax8*^*rtTA*^/*TRE*^*ICNotch1*^ mice (S2 Table), with lower expression in Notch transgenic animals. We found that RBPJ could directly bind to the *Egf* regulatory region (S7A Fig). Previous studies have identified a correlation between kidney or urinary *EGF* transcript with fibrosis or kidney function decline [46,47]. We also found that *EGF* showed a strong negative correlation with kidney fibrosis score in human kidney samples (*P* = 8.91 × 10^−7^) (S7B Fig). *Egf* mRNA was consistently lower in kidneys of FA, UUO, and APOL1 CKD mouse models (S7C–S7E Fig). To understand the role of EGF, we added Recombinant Human EGF to the culture medium. We found that exogenous EGF was unable to ameliorate Notch-induced expression of *Acta2* and *Snai1* (S7F and S7G Fig). In summary, we were unable to connect the Notch-induced *Egf* changes to fibrosis development.

From the list of direct target genes, we next focused on *Tfam*. *Tfam* is one of the key transcriptional regulators of mitochondrial (and therefore metabolic) gene expression. ChIP-seq analysis indicated RBPJ binding to the *Tfam* regulatory region (at chr10: 70700475–70701075) (Fig 6B). *TFAM* was not only down-regulated directly by Notch signaling, but its expression was also negatively associated with the degree of kidney fibrosis in human patient kidney samples (*P* = 0.05) (Fig 6C). *Tfam* expression was lower not only in tubule-specific Notch transgenic mice but also in the UUO and APOL1-induced kidney fibrosis models (Fig 6D and 6E).

To understand whether reduced *Tfam* expression plays a role in tubule epithelial dedifferentiation, we generated a new mouse model with tubule-epithelial–specific deletion of *Tfam* by mating the *Tfam*^*flox*/*flox*^ and *Ksp*^*Cre*^ mice (S8A and S8B Fig). Ksp^cre^/Tfam^flox/flox^ mice were born at expected Mendelian distribution. We found fewer than expected (around 50% fewer) *Ksp*^*Cre*^/*Tfam*^*flox*/*flox*^ mice in the litters when compared to heterozygous or wild-type CTLs by weaning age (postnatal day 20, P20) (Table 3). By postnatal day 15, surviving animals were significantly smaller than CTL littermates and died by 15 weeks of age (S8C Fig). Histological examination indicated mitochondrial alterations in tubule epithelial cells of *Ksp*^*Cre*^/*Tfam*^*flox*/*flox*^ mice, indicating the key role of *Tfam* in renal tubule homeostasis and metabolism (Fig 6F). We found that *Ksp*^*Cre*^/*Tfam*^*flox*/*flox*^ mice had profibrotic gene expression changes as indicated by higher levels of *Fibronectin*, *Vimentin*, *Collagen1*, *3*, and *Acta2* on P20 (Fig 6G). Overall, we found a direct binding of RBPJ into the *Tfam* promoter and reduced *Tfam* expression following Notch activation. *Tfam* expression in tubule cells was critically important for kidney function, as mice with genetic deletion of *Tfam* in tubule cells were sufficient to induce fibrosis.

**Table 3:** Genotypes obtained after mating *Ksp*^*cre*^ *Tfam* ^flox/w*t*^ (male) and *Tfam* ^flox/flo*x*^ (female) mice.

Stage	Offspring (*n*)	*Tfam*^flox/wt^	*Tfam*^flox/flox^	*Ksp*^*cre*^ *Tfam*^flox/wt^	*Ksp*^*cre*^*Tfam*^flox/flox^
E13.5–E18.5	19	3 (16%)	5 (26%)	6 (32%)	5 (26%)
Newborn	22	6 (27%)	5 (23%)	5 (23%)	6 (27%)
P20	63	19 (30%)	20 (32%)	17 (27%)	7 (11%)

**Abbreviation:** E13.5, embryonic day 13.5; E18.5, embryonic day 18.5; P20, postnatal day 20.

### *Tfam* can rescue Notch-induced metabolic defect and downstream profibrotic changes

**Fig 7:**
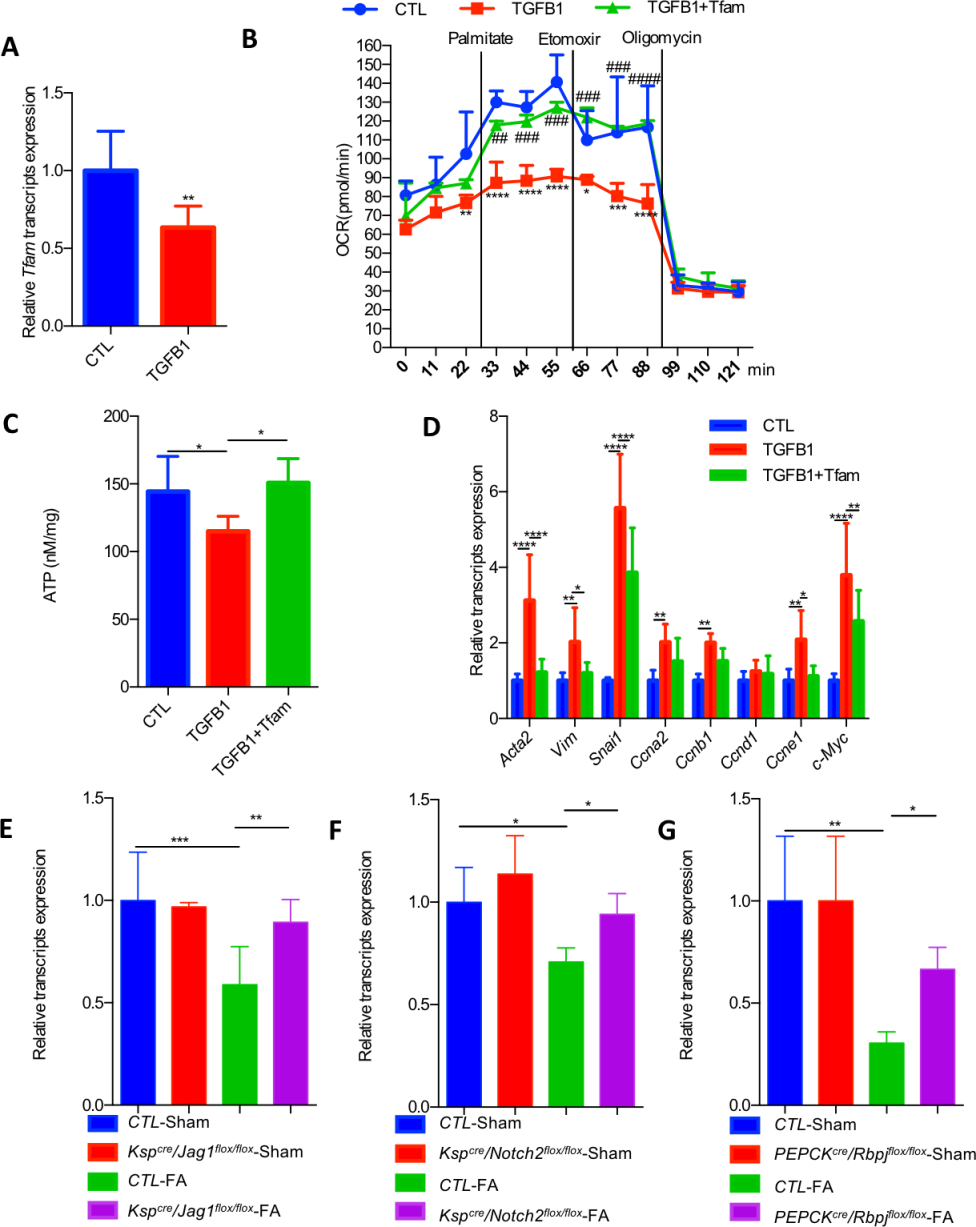
*Tfam* rescues TGF-β1/Jag1/Notch2-induced metabolic defect and downstream profibrotic changes. (A) *Tfam* expression level in NRK-52E cells treated with TGF-β1 for 24 h. Data are represented as mean ± SD. ** *P* < 0.01 by two-tailed Student *t* test (*n* = 8 per group). (B) OCR in NRK-52E cells exposed to 5 ng/ml TGF-β1 for 24 h in the presence of GFP or *TFAM* plasmid. Where indicated, cells were incubated in palmitate (180 μM), etomoxir (40 μM), and oligomycin (1 μM). Data are represented as mean ± SD. * *P* < 0.05, **P < 0.01, *** *P* < 0.001, and **** *P* < 0.0001 as compared to CTL group, ## *P* < 0.01, ### *P* < 0.001, and #### *P* < 0.0001 as compared to TGF-β1 group by two-way ANOVA with post hoc Tukey test (*n* = 3 per group). (C) ATP levels in NRK-52E cells and cells treated with TGF-β1 for 24 h in the presence of GFP or *TFAM* plasmid. Data are represented as mean ± SD. * *P* < 0.05 by one-way ANOVA with post hoc Tukey test (*n* = 8, 6, 6). (D) Relative mRNA expression of transcripts related to fibrosis, dedifferentiation, and proliferation in NRK-52E cells treated with or without TGF-β1 and transfected with GFP or *TFAM* plasmids. Data are represented as mean ± SD. * *P* < 0.05, ** *P* < 0.01, and **** *P* < 0.0001 by two-way ANOVA with Tukey post hoc tests (*n* = 8, 8, 7). (E) Relative mRNA amount of *Tfam* in CTL and *Ksp*^*cre*^*Jag1*^*flox*/*flox*^ mice with or without FA injection. Data are represented as mean ± SD. ** *P* < 0.01 and *** *P* < 0.001 by one-way ANOVA with post hoc Tukey test (*n* = 5, 3, 9, 10). (F) Relative mRNA amount of *Tfam* in CTL and *Ksp*^*cre*^/*Notch2*^*flox*/*flox*^ mice with or without FA injection. Data are represented as mean ± SD. * *P* < 0.05 by one-way ANOVA with post hoc Tukey test (*n* = 5, 6, 5, 7). (G) Relative mRNA amount of *Tfam* in CTL and *PEPCK*^*cre*^/*Rbpj*^*flox*/*flox*^ mice with or without FA injection. Data are represented as mean ± SD. * *P* < 0.05 and ** *P* < 0.01 by one-way ANOVA with post hoc Tukey test (*n* = 3, 3, 5, 6). The underlying data of panels A–G can be found in S1 Data. CTL, control; FA, folic acid; GFP, green fluorescent protein; OCR, oxygen consumption rate; Tfam, mitochondrial transcription factor A.

As we identified *Tfam* as a direct Notch target, we hypothesized that the widespread effect of Notch on gene expression and RTEC dedifferentiation is mediated by direct metabolic reprogramming. To understand the functional consequences of Notch-induced reduction of *Tfam* expression, we have examined mitochondrial function in RTECs. RTECs exclusively rely on mitochondrial fatty acid oxidation (FAO) as their energy source [48,49]. First, we examined *Tfam* expression in vitro following *Jag1*/*Notch2* activation. *Tfam* expression was lower in RTECs treated with TGF-β1 and also in the presence of JAG1-expressing cells (Fig 7A; S9A Fig). We found that higher Notch activation was associated with lower FAO in renal tubule cells. Increasing *Tfam* expression in RTECs improved the FAO defect both in the TGF-β1–induced Notch activation models and in the JAG1 co-culture system (Fig 7B and 7C; S9B and S9C Fig), thus indicating that *Tfam* is functionally important in mediating the Notch-induced metabolic reprogramming.

Furthermore, the improved FAO and mitochondrial content significantly ameliorated the Notch-induced profibrotic gene expression changes (Fig 7D; S9D Fig). We also found that transfection of tubule cells with *Tfam* reduced the expression of mesenchymal cell markers, such as *Acta2* and *Snail1*, and markers of proliferations such as *cyclins* even in absence of TGF-β1. These results indicated that in vitro cultured RTECs likely need higher mitochondrial activity to maintain their epithelial characteristics in vitro (S10A Fig). To prove that the rescue effect was related to improved FAO, we have also treated cells with the peroxisome proliferator-activated receptor α (PPARα) agonist, fenofibrate. We found that fenofibrate could rescue Jag1-induced fibrosis (S10B–S10D Fig), indicating the key role of metabolic changes in Notch-induced fibrosis.

**Fig 8:**
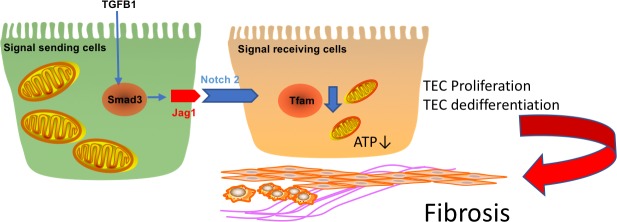
Tubule-specific *Jag1*/*Notch2* signaling plays a key role in kidney fibrosis development by regulating metabolism via *Tfam*. TEC, tubular epithelial cells; Tfam, mitochondrial transcription factor A.

Finally, we have examined whether *Jag1* and *Notch2* deletion can rescue mice from the metabolic defect observed in mouse kidney fibrosis samples. We found that *Tfam* expression was lower in the FA-induced kidney fibrosis in CTL groups. Genetic deletion of *Jag1* and *Notch2* prevented the decrease in *Tfam* expression and downstream fibrosis development (Fig 7E and 7F**)**. On the other hand, mice with genetic deletion of *Notch1* and *Notch3* were not protected from fibrosis-induced decrease in *Tfam* expression (S9E and S9F Fig). We also analyzed *Tfam* expression in proximal tubule-specific RBPJ knockout mice (*PEPCK*^*cre*^/*Rbpj*^*flox*/*flox*^) with or without FA injection. We used this model in our previous paper and showed a protection from FA-induced fibrosis, as RBPJ is a common transcriptional coactivator in Notch signaling [8]. *Tfam* expression was lower in the FA model, which was ameliorated in absence of RBPJ, indicating that *Tfam* is a RBPJ target (Fig 7G). In summary, these results strongly indicate that the Notch-induced inhibition of *Tfam* and downstream metabolic reprogramming are responsible for the profibrotic effect of Notch both in vivo and in vitro (Fig 8).

## Discussion

Here, we showed that the Notch ligand *Jag1* and the Notch receptor *Notch2* expression in renal tubule cells play a key role in kidney fibrosis development. While previous studies from our and other groups have already suggested the contribution of Notch signaling in CKD, most prior studies have used models with global inhibition or activation of Notch signaling [8,50,51]. While such studies were instrumental to the understanding of kidney fibrosis biology, they did not represent clinically translatable strategies, as long-term systemic inhibition of Notch signaling is associated with significant side effects [20]. Systemic Notch inhibition by gamma secretase inhibitors are associated with significant intestinal toxicity, as Notch signaling plays a key role in intestinal cell differentiation. To circumvent these issues, receptor- and ligand-isoform–specific targeting is emerging with improved side-effect profiles.

Here, we used mice with tubule-specific genetic deletion of *Notch1* and *Notch2* and global deletion of *Notch3*, and we show that only mice with tubule-specific loss of *Notch2* are protected from kidney fibrosis. Our previous work showed that tubule-specific NICD1 gain of function was sufficient to induce fibrosis, indicating that Notch1 gain of function can likely substitute for other Notch isoforms. Similarly, in vitro overexpression of either intracellular domain of NOTCH1 (ICN1) or NOTCH2 (ICN2) was sufficient to induce profibrotic gene expression changes [8]. These results are less surprising, as the intracellular domain of the Notch receptors are highly homologous. Genetic loss of *Jag1* in tubule epithelial cells protected animals from fibrosis development. These results are consistent with unbiased gene expression analysis results that indicated that the expression of *JAG1* and *NOTCH2* were the highest in mouse and human kidneys. The expression of *JAG1* and *NOTCH2* showed the strongest correlation with kidney fibrosis in human kidneys. In addition to our current direct genetic models, several other prior studies suggested Notch2 as an indirect target of pharmaceutical interventions for kidney fibrosis. For example, the Lorenzen group showed that miR-21 is an important upstream regulator of *Notch2*, and therefore the miR-21 antagonist that is currently in clinical studies might also reduce *Notch2* activation [52]. Similar studies showed that Klotho deficiency can cause fibrosis via *Notch2* activation [53]. It is important to note that isoform-specific Notch-receptor targeting has been more difficult to develop than ligand targeting; furthermore, one study examining the role of *Notch2* in podocytes in nephrotic syndrome indicated some potential benefit [54]. Therefore, ligand targeting such as JAG1 appears to be the most reasonable approach for CKD. Of note, the lateral aspect of RTECs with the higher expression of JAG1 and NOTCH2 is likely the site for interaction in fibrosis, which is similar to Notch activation in development stage. Our studies can provide the foundation for the development of novel molecular therapeutics for kidney failure and advance our understanding of the renal effect of JAG1 antagonism-based therapies.

Our studies show that Notch directly binds to the metabolic transcription factor *Tfam* to reprogram tubule epithelial metabolism and induces tubule epithelial dedifferentiation and proliferation. While prior work indicated the role of metabolic changes in kidney disease development, here we show a direct interaction between Notch and metabolic changes via direct targeting *Tfam*. Kidney tubules are highly metabolic cells, and many prior studies have focused on identification of the key metabolic transcription factor for tubule cells. Studies have demonstrated a decrease in expression of *Ppargc1a*, *Ppara*, and *Pparg* in diseased kidneys [55–58]. Transgenic expression of *Ppargc1a* and *Ppara* showed protection from acute kidney injury and kidney fibrosis and also rescued the phenotype of the tubule-specific Notch transgenic mice, indicating the key role of metabolic pathways in kidney function and tubule health [48,58,59]. On the other hand, mice with global deletion of *Ppargc1a* and *Ppara* did not present with renal abnormalities, thus raising significant doubts that the reduced expression of these transcription factors alone are sufficient for fibrosis development [58,60]. Here, we show that *Tfam* is the essential transcription factor for RTEC metabolism. Tubule-specific deletion of *Tfam* in mice results in tubule epithelial dedifferentiation and fibrosis. These results indicate that while transgenic expression of *Ppargc1a* and *Ppara* showed protection, the key metabolic transcription factor in the kidney is *Tfam*, as no other metabolic transcription factors (like *Ppara*, *Pparg*, etc.) were able to compensate for its loss.

We have also analyzed other potential Notch targets. We found a direct Notch binding to the *Egf* promoter and subsequent inverse correlation between Notch levels and EGF expression in the kidney. Our in vitro studies failed to establish a causal relationship between Notch-regulated *Egf* levels and subsequent kidney fibrosis development. Overall, our studies indicate that Notch-induced global changes in differentiation were mediated by the regulation of cellular metabolism rather than individual-targeting individual-differentiation–associated gene expression. Given the broad transcriptional program Notch needs to regulate, changing cellular metabolism could become a key component to alter cell differentiation.

Future studies shall aim to understand the mechanism of sustained and excessive Notch activation in CKD. Tonic Notch activation is likely important in orchestrating the regenerative response including transit amplification and proliferation. It seems, however, that in CKD, Notch activation is excessive and sustained, which might be caused by the sustained epithelial injury. Overall, our studies indicate that targeting the specific Notch ligand JAG1 or receptor NOTCH2 could have important therapeutic potential for the treatment of CKD.

## Materials and methods

### Ethics statement

The human subject study was approved by the institutional review boards (IRBs) of the Albert Einstein College of Medicine and Montefiore Medical Center (IRB 2002–202) and the University of Pennsylvania (IRB 815796). All experiments on animals were reviewed and approved by the Institutional Animal Care and Use Committee of the University of Pennsylvania (Protocol #804141) and were performed in accordance with the institutional guidelines.

### Mice

For FA and UUO mouse models, 6- to 8-week-old male FvB wild-type mice were used. Mice were injected with FA (250 mg/kg, dissolved in 300 mM sodium bicarbonate) intraperitoneally (ip) and killed on day 7, and sodium-bicarbonate–injected mice were used as CTLs. For the UUO model, mice underwent ligation of the left ureter and were killed on day 7, and sham-operated mice were used as the CTL group. For APOL1 mouse model, 4- to 6-week-old male FvB transgenic mice (*Nphs1*-rtTA/TRE-*APOL1*-G2) were placed on a doxycycline diet to induce transgene expression as described previously [41]. Mice with *Jag1*-floxed alleles were kindly provided from Dr. Verdon Taylor. Mice with a floxed *Notch1* allele were purchased from Jackson Lab (stock #006951). Mice with *Notch2* floxed allele were a gift from Dr. Ursula Zimber-Strobl. *Tfam^floxed^* mice were purchased from Jackson Lab (stock #026123). *Jag1*^*flox*/*flox*^, *Notch1*^*flox*/*flox*^, *Notch2*^*flox*/*flox*^, and *Tfam*^*flox*/*flox*^ mice were crossed with transgenic mice expressing Cre recombinase under the cadherin 16 promoter (*Ksp-Cre*) (Jackson Lab, stock #012237). Single- or double-floxed mice without *Ksp*–*Cre* served as the CTL group (WT/Jag1^flox/flox^ mice and WT/Jag1^flox/+^ mice for tubule-specific Jag1 deletion mice, WT/Notch2^flox/flox^ mice for tubule-specific Notch2 deletion mice, WT/Notch1^flox/flox^ mice, and WT/Notch1^flox/+^ mice for tubule-specific Notch1 deletion mice). *Notch3* knockout mice were purchased from Jackson Lab (stock #010547). PEPCK^cre^/Rbpj^flox/flox^ mice were generated in our previous paper [8]. 6- to 8-week-old male mice were ip injected with 250 mg/kg FA in 300 mM sodium bicarbonate and killed on day 7 post.

### qRT-PCR

RNA was isolated from harvested kidney tissues and cells using Trizol (Invitrogen). RNA (1 μg) was reverse transcribed using the cDNA Archival Kit (Life Technologies), and qRT-PCR was run in the ViiA 7 System (Life Technologies) instrument using SYBR Green Master Mix and gene-specific primers. The data were normalized and analyzed using the ΔΔCt method. The primers used are listed in S3 Table. The species-specific primers were previously published and confirmed by primer-blast search on NCBI [8,36,61].

### Gene expression analysis

FA nephropathy model: male Balb/c mice (25–29 g) received a single ip injection of 250 mg/kg FA dissolved in a 300 mM sodium bicarbonate solution. Mice were killed at 3 days following administration. RNA sequencing data from FA treatment mouse kidney samples were compared with normal group (GSE65267, GSM1591197-GSM1591199 versus GSM1591206-GSM1591208) [39]. UUO model: 8- to 12-week-old male C57BL/6 mice were sham-operated or underwent ureter ligation for 8 days. UUO mice were compared with their sham-operated group (GSE79443, GSM2095449-GSM2095452 versus GSM2095456-GSM2095458) [40]. APOL1 model: 4- to 6-week-old male FvB transgenic mice (WT, *Nphs1*-rtTA/TRE-*APOL1*-G0, *Nphs1*-rtTA/TRE-*APOL1*-G1, and *Nphs1*-rtTA/TRE-*APOL1*-G2) mice were placed on a doxycycline diet to induce transgene expression as described previously [41]. Mice with transgenic expression of APOL1 risk variants (G1 and G2) in podocytes were compared with CTL littermates (GSE81492, GSM2154804-GSM2154809 versus GSM2154800-GSM2154803).

### In situ hybridization

In situ hybridization was performed using formalin-fixed paraffin-embedded tissue samples and the RNAscope 2.5 HD Duplex Detection Kit (ACDBio #322436). We followed the manufacturer’s protocol. Mm-*Jag1* (ACDBio #412831) and Mm-*Notch2*-C2 (ACDBio #425161-C2) probes were used for the RNAscope assay. *Jag1* and *Notch2* signals were extracted out for quantification using color deconvolution by Fiji[62]. For each section, five random fields were quantified in an unbiased manner using ImageJ.

### Staining

Kidneys were harvested from CTL and FA-injected mice or human kidneys. Histological analysis was examined on formalin-fixed, paraffin-embedded kidney sections stained by PAS and Picrosirius red (Polyscience #24901). Immunofluorescence staining was performed with antibodies against JAG1 (Abcam #ab109536; 1:500) or NOTCH2 (Abcam #ab8926; 1:100). Tubule specific markers were used as previously described [18,63]. Immunohistochemistry staining was performed with antibody against αSMA (Sigma #A5228; 1:500) and COX IV (Abcam # ab16056; 1:200) in paraffin-embedded kidney sections. For each section, five random fields were quantified in an unbiased manner using ImageJ.

### Human samples

95 tubule compartments of human kidney samples were microdissected from nephrectomies as previously described (ArrayExpress: E-MTAB-5929, E-MTAB-2502) [42]. Fibrosis scores for all samples were quantified by a pathologist using PAS-stained kidney sections. Correlation between fibrosis score and gene expression used a linear regression model adjusted for gender, age, and race. The statistics analysis was performed with the R software package (v3.2.2), and the model is lm (Gene Expression ~ Fibrosis + Gender + Age + Race). We have reported beta value (effect size) and *P*-value (significance) obtained from this regression model.

### Western blot

Cell lysates were prepared with SDS lysis buffer containing protease inhibitor cocktail (cOmplete Mini, Roche #11836153001) and phosphatase inhibitor (PhosSTOP, Roche #4906837001). Proteins were resolved on 8%–12% gradient gels, transferred on to polyvinylidene difluoride membranes, and probed with antibodies as below: JAG1 (Abcam #ab109536; 1:1000), NOTCH2 (Abcam #ab8926; 1:500), TFAM (Millipore #ABS2083; 1:1000), and β actin (Abcam #ab8226; 1:5000). Anti-rabbit (Cell Signaling #7074; 1:5000) or anti-mouse (Cell Signaling #7076; 1:5000) IgG horseradish peroxidase was used as a secondary antibody. Blots were detected by enhanced chemiluminescence (Western Lightning-ECL, Thermo Scientific).

### BUN and creatinine level

Serum creatinine and BUN were determined by Mouse Creatinine Kit (Crystal Chem #80350) and TRACE DMA Urea kit (Thermo Electron Corporation #TR12003), respectively, according to the manufacturers’ instructions.

### Primary culture of renal tubule cells

Primary culture of mouse renal epithelial cell and TGF-β1 treatment was done as previously described [48]. Kidneys were collected from FvB wild-type mice (3- to 5-week-old males). Cells were isolated by 2 mg/ml collagenase I (Worthington Biochemical Product #CLS-1) digestion for 30 min at 37°C with gentle stirring. Cells were then filtered through the 100-mm mesh to isolate single cells. Cell suspensions were cultured in RPMI 1640 (Corning #10-040-CM) supplement with 10% fetal bovine serum (FBS; Atlanta Biologicals #S11950), 20 ng/ml EGF (Peprotech #AF-100-15), 1× ITS (Gibco #51500–056), and 1% penicillin-streptomycin (Corning #30-002-CI) at 5% CO2 and 37°C. When cell confluence reached 70%, the media were changed with serum-free RPMI for 24 h. After that, cells were treated with 50 ng/ml TGF-β1 (Peprotech #100–21) for 24 h.

### RNA sequencing

RNA quality was assessed with the Agilent Bioanalyzer 2100, and only samples with RIN scores above 7 were used for cDNA production. RNA (100 ng) was used to isolate poly(A)-purified mRNA using the Illumina TruSeq RNA Preparation Kit. Single-end 50-bp sequencing was performed in an Illumina HiSeq, and the annotated RNA counts (FASTQ) were calculated by Illumina’s CASAVA 1.8.2. Sequence quality was surveyed with FastQC. Adaptor and lower-quality bases were trimmed with TrimGalore. Reads were aligned to the Gencode mouse genome (GRCm38) using STAR-2.4.1d. Read counts for each sample were obtained using HTSeq-0.6.1 (htseqcount). RNA sequencing data has been deposited in the Gene Expression Omnibus (GEO) with the accession code GSE118600.

### Cell culture

Rat epithelial cells (NRK-52E) from ATCC (CRL-1571) were cultured in DMEM with 5% FBS. L cells and JAG1-expressing cells were a gift from Dr. Pamela Stanley from the Albert Einstein College of Medicine. For co-culture system, NRK-52E cells were firstly plated, then incubated overnight and reached 70%–80% confluence the next day. Then, L cells and JAG1-expressing L cells were plated on top of signal-receiving cells at a density equal to the monolayer to establish a direct contact between ligand and receptor. The cells were co-cultured in growth medium for 24 h. For the rescue experiment, NRK-52E were either transiently cotransfected with *TFAM*-expressing plasmid and GFP as CTL or EGF. Transfection efficiency was confirmed by GFP visualization. Following transfection, the cells were co-cultured with either JAG1-expressing L cells or L cells for 24 h or were treated with TGF-β1 (5 ng/ml) for 48 h. pCellFree_G03 *TFAM* was a gift from Dr. Kirill Alexandrov (Addgene plasmid #67064) [64]. Recombinant Human EGF was purchased from Peprotech (#AF-100-15). DBZ was purchased from CalBiochem (#565789).

### Cell proliferation assay

A BrdU-based cell proliferation assay was performed on JAG1 co-culture system according to the manufacturer’s instructions (EMD Millipore #2750).

### ChIP-seq

RBPJ ChIP-seq dataset with 6 h and 24 h Notch activation and inhibition was adapted from cultured cells (GSE37184) [45].

### Oxygen consumption experiment

FAO was studied using SeaHorse Bioscience metabolic analyzer as previously described [48]. We followed the manufacturer’s instructions to monitor oxygen consumption rate (OCR) in TGF-β1-treated NRK52E cells or co-culture system. Briefly, cells were seeded in XF24 V7 cell culture microplate at 2 × 10^4^ cells per well. OCR was assessed at baseline and after the addition of palmitate-conjugated BSA (180 μM) followed by the addition of the carnitine palmitoyltransferase-1 inhibitor etomoxir (40 μM). The final state was determined after the addition of the ATP synthase inhibitor oligomycin (1 μM).

### ATP measurement

The ATP concentration of cultured cells was measured using an ATP Fluorometric Assay Kit (BioVision #K354) according to the manufacturer’s protocol.

### Statistics

Statistical analyses were performed using GraphPad Prism 6.0 software. All values are expressed as mean and standard deviation. A two-tailed Student *t* test was used to compare 2 groups. One-way ANOVA or two-way ANOVA with post hoc Tukey test was used to compare multiple groups. A *P*-value less than 0.05 was considered statistically significant.

## Supporting information

S1 Data Underlying data for Figs 1–7, S1–S7 Figs, S9 Fig and S10 Fig. (XLSX)

S1 Table RBPJ binding sites overlap with up-regulated genes in *Pax8*^*rtTA*^/*TRE*^*ICNotch1*^ mice. RBPJ, recombination signal binding protein for immunoglobulin kappa J region.
(DOC)

S2 Table RBPJ binding sites overlap with down-regulated genes in *Pax8*^*rtTA*^/*TRE*^*ICNotch1*^ mice. RBPJ, recombination signal binding protein for immunoglobulin kappa J region.
(DOC)

S3 Table qPCR primer sequences. (DOC)

S1 Fig Increased *Jag1*/*Notch2* expression in mouse kidney fibrosis. (A–C) Normalized counts of Notch ligands and receptors by RNA sequencing in whole-kidney lysates of FA-induced nephropathy group (*n* = 3 per group) (A), sham and UUO group (*n* = 4, 3) (B), and APOL1-G1/G2 mice (*n* = 6, 4) (C). Data are represented as the mean ± SD. * *P* < 0.05 and ** *P* < 0.01 by two-tailed Student *t* test. (D and E) Representative in situ hybridization images of *Jag1* (D) and *Notch2* (E) on sham and FA-treated mouse kidneys. Scale bar: 10 μm. (F and G) Relative *Jag1* (F) and *Notch2* **(**G) in situ hybridization intensity on sham and FA-treated mouse kidneys. ** *P* < 0.01 by two-tailed Student *t* test (*n* = 3 per group). (H and I) Relative JAG1 (H) and NOTCH2 (I) immunofluorescence intensity on sham or FA-treated mouse kidneys. ** *P* < 0.01 and **** *P* < 0.0001 by two-tailed Student *t* test (*n* = 4 per group). The underlying data of panels A, B, C, F, G, H, and I can be found in S1 Data. APOL1-G1/G2, apolipoprotein L1-G1 and G2 risk alleles; FA, folic acid; N/A, not available; UUO, unilateral ureteral obstruction.
(TIFF)

S2 Fig Reduced fibrosis in tubule-specific *Jag1* knockout mice. (A) Experimental scheme for generating the *Ksp*^*cre*^/*Jag1* ^*flox*/*flox*^ mice. Kidney injury was induced by FA injection. (B) Western blot analysis of JAG1 in whole kidney lysates of CTL and *Ksp*^*cre*^/*Jag1*^*flox*/*flox*^ mice. β-actin was used as a loading CTL. (C) Quantification of Sirius-Red–stained kidney sections from CTL and *Ksp*^*cre*^ /*Jag1*^*flox*/*flox*^ mice with or without FA injection. Data are represented as mean ± SD. *** *P* < 0.001 and **** *P* < 0.0001 by one-way ANOVA with post hoc Tukey test (*n* = 3 per group). (D) Quantification of αSMA-stained kidney sections from CTL and *Ksp*^*cre*^/*Jag1*^*flox*/*flox*^ mice with or without FA injection. Data are represented as mean ± SD. ** *P* < 0.01 by two-tailed Student *t* test (*n* = 3 per group). The underlying data of panels C and D can be found in S1 Data. αSMA, alpha smooth muscle actin; CTL, control; FA, folic acid.
(TIFF)

S3 Fig Reduced fibrosis in tubule-specific *Notch2* knockout mice. (A) Experimental scheme for generating the *Ksp*^*cre*^/*Notch2* ^*flox*/*flox*^ mice. Kidney injury was induced by FA injection. (B) Western blot analysis of NOTCH2 in whole kidney lysates of CTL and *Ksp*^*cre*^/*Notch2*^*flox*/*flox*^ mice. β-actin was used as a loading CTL. (C) Quantification of Sirius-Red–stained kidney sections from CTL and *Ksp*^*cre*^ /*Notch2*^*flox*/*flox*^ mice with or without FA injection. Data are represented as mean ± SD. *** *P* < 0.001 and **** *P* < 0.0001 by one-way ANOVA with post hoc Tukey test (*n* = 3 per group). (D) Quantification of αSMA-stained kidney sections from CTL and *Ksp*^*cre*^/*Notch2*^*flox*/*flox*^ mice with or without FA injection. Data are represented as mean ± SD. *** *P* < 0.001 by two-tailed Student *t* test (*n* = 3 per group). The underlying data of panels C and D can be found in S1 Data. αSMA, alpha smooth muscle actin; CTL, control; FA, folic acid.
(TIFF)

S4 Fig No change in fibrosis development in tubule-specific *Notch1* knockout mice. (A) Experimental scheme for generating the *Ksp*^*cre*^/*Notch1* ^*flox*/*flox*^ mice. Kidney injury was induced by FA injection. (B) Representative images of PAS-stained kidney sections from CTL and *Ksp*^*cre*^ /*Notch1*^*flox*/*flox*^ mice with or without FA injection. Scale bar: 10 μm. (C and D) Relative mRNA amount of Notch signaling (C) and fibrosis markers (D) in CTL and *Ksp*^*cre*^/*Notch1*^*flox*/*flox*^ mice with or without FA injection. Data are represented as mean ± SD. * *P* < 0.05, ** *P* < 0.01, *** *P* < 0.001, and **** *P* < 0.0001 by two-way ANOVA with post hoc Tukey test (*n* = 7, 5, 6, 5). The underlying data of panels C and D can be found in S1 Data. CTL, control; FA, folic acid; PAS, Periodic acid–Schiff.
(TIFF)

S5 Fig No change in kidney fibrosis development in *Notch3* knockout mice. (A) Experimental scheme for generating the *Notch3* knockout mice. Kidney injury was induced by FA injection. (B) Representative images of PAS-stained kidney sections from CTL and *Notch3* knockout mice with or without FA injection. Scale bar: 10 μm. (C and D) Relative mRNA amount of Notch signaling (C) and fibrosis markers (D) in CTL and *Notch3* knockout mice with or without FA injection. Data are represented as mean ± SD. * *P* < 0.05, *** *P* < 0.001, and **** *P* < 0.0001 by two-way ANOVA with post hoc Tukey test (*n* = 6, 5, 4, 4). The underlying data of panels C and D can be found in S1 Data. CTL, control; FA, folic acid; PAS, Periodic acid–Schiff.
(TIFF)

S6 Fig Characterization of the JAG1 co-culture system. (A) Expression of m*Jag1* in JAG1-expressing L cells compared to CTL L cells. Data are represented as the mean ± SD. **** *P* < 0.0001 by two-tailed Student *t* test (*n* = 12 per group). (B) Relative mRNA amount of *Hes1* and *Hey1* in CTL and JAG1-expressing L cell with or without DBZ. Data are represented as mean ± SD. * *P* < 0.05, ** *P* < 0.01, and **** *P* < 0.0001 by two-way ANOVA with post hoc Tukey test (*n* = 8, 4, 8, 4). The underlying data of panels A and B can be found in S1 Data. CTL, control; DBZ, dibenzazepine.
(TIFF)

S7 Fig EGF did not rescue JAG1-induced metabolic defect and downstream profibrotic changes. (A) The mouse *Egf* locus and RBPJ ChIP-Seq from 6 h and 24 h Notch activation and inhibition. (B) Correlation between interstitial fibrosis and *EGF* transcript level in 95 microdissected human kidney samples. (C–E) Normalized counts of *Egf* by RNA sequencing in whole-kidney lysates of FA-induced nephropathy group (*n* = 3 per group) (C), sham and UUO group (*n* = 4, 3) (D), and APOL1-G1/G2 mice (*n* = 6, 4) (E). Data are represented as the mean ± SD. *** *P* < 0.001 and **** *P* < 0.0001 by two-tailed Student *t* test. (F and G) Relative mRNA expression of *Acta2* (F) and *Snai1* (G) in JAG1 co-culture system when treated with EGF. Data are represented as mean ± SD. * *P* < 0.05 and ** *P* < 0.01 by one-way ANOVA with post hoc Tukey test (*n* = 8, 7, 4, 4). The underlying data of panels B, C, D, E, and F can be found in S1 Data. APOL1-G1/G2, apolipoprotein L1-G1 and G2 risk alleles; EGF, epidermal growth factor; FA, folic acid; RBPJ, recombination signal binding protein for immunoglobulin kappa J region; UUO, unilateral ureteral obstruction.
(TIFF)

S8 Fig *Tfam* plays an important role in kidney tubules. (A) Experimental scheme for generating the *Ksp*^*cre*^/*Tfam*^*flox*/*flox*^ mice. (B) Western blot analysis of TFAM in whole kidney lysates of *WT*/*Tfam*^*flox*/*flox*^, *Ksp*^*cre*^/*Tfam*^*flox*/*wt*^ and *Ksp*^*cre*^/*Tfam*^*flox*/*flox*^ mice. β-actin was used as a loading CTL. (C) Appearance of *WT*/*Tfam*^*flox*/*flox*^ and *Ksp*^*cre*^/*Tfam*^*flox*/*flox*^ pups at postnatal day 15. Scale bar: 1 cm. CTL, control; Tfam, mitochondrial transcription factor A.
(TIFF)

S9 Fig *Tfam* rescues JAG1-induced metabolic defect and downstream profibrotic changes. (A) *Tfam* expression level in JAG1 co-culture system. Data are represented as mean ± SD. ** *P* < 0.01 by two-tailed Student *t* test (*n* = 6 per group). (B) OCR in JAG1 co-culture system in the presence of *GFP* or *TFAM* plasmid. Where indicated, cells were incubated in palmitate (180 μM), etomoxir (40 μM), and oligomycin (1 μM). Data are represented as mean ± SD. *P < 0.05, ** *P* < 0.01, *** *P* < 0.001, and **** *P* < 0.0001 as compared to L cell group, # *P* < 0.05, ## *P* < 0.01, and ### *P* < 0.001 as compared to JAG1 cell group by two-way ANOVA with post hoc Tukey test (*n* = 3 per group). (C) ATP levels in JAG1 co-culture system in the presence of *GFP* or *TFAM* plasmid. Data are represented as mean ± SD. * *P* < 0.05 by one-way ANOVA with post hoc Tukey test (*n* = 8, 6, 6). (D) Relative mRNA expression of transcripts related to dedifferentiation and proliferation in JAG1 co-culture system in the presence of GFP or *TFAM* plasmid. Data are represented as mean ± SD. * *P* < 0.05 and **** *P* < 0.0001 by one-way ANOVA with post hoc Tukey test (*n* = 8 per group). (E) Relative mRNA amount of *Tfam* in CTL and *Ksp*^*cre*^/*Notch1*^*flox*/*flox*^ mice with or without FA injection. Data are represented as mean ± SD. * *P* < 0.05 by one-way ANOVA with post hoc Tukey test (*n* = 7, 5, 6, 5). (F) Relative mRNA amount of *Tfam* in CTL and *Notch3* knockout mice with or without FA injection. Data are represented as mean ± SD. * *P* < 0.05 by one-way ANOVA with post hoc Tukey test (*n* = 6, 5, 4, 4). The underlying data of panels A–F can be found in S1 Data. CTL, control; FA, folic acid; GFP, green fluorescent protein; OCR, oxygen consumption rate; Tfam, mitochondrial transcription factor A.
(TIFF)

S10 Fig PPARα agonist rescues JAG1 induced dedifferentiation. (A) Relative mRNA expression of transcripts related to fibrosis, dedifferentiation, and proliferation in NRK52E cells transfected with *TFAM* plasmids. Data are represented as mean ± SD. * *P* < 0.05, ** *P* < 0.01, and *** *P* < 0.001 by two-tailed Student *t* test (*n* = 6 per group). (B) Relative mRNA expression of *Ppara* and *Ppargc1a* in JAG1 co-culture system. Data are represented as mean ± SD. **** *P* < 0.0001 by two-tailed Student *t* test (*n* = 6 per group). (C and D) Relative mRNA expression of *Acta2* (C) and *Snai1* (D) in JAG1 co-culture system when treated with Feno. Data are represented as mean ± SD. ** *P* < 0.01 and **** *P* < 0.0001 by one-way ANOVA with post hoc Tukey test (*n* = 8, 7, 4, 4). The underlying data of panels A and B can be found in S1 Data. CTL, control; Feno, fenofibrate; PPARα, peroxisome proliferator-activated receptor α.
(TIFF)

## Abbreviations

APOL1-G1/G2: apolipoprotein L1-G1 and G2 risk alleles
AQP2: aquaporin 2
αSMA: alpha smooth muscle actin
ATP6V1B1: ATPase H+ Transporting V1 Subunit B1
BrdU: bromodeoxyuridine
BUN: blood urea nitrogen
ChIP-seq: chromatin immunoprecipitation sequencing
CKD: chronic kidney disease
COX IV: cytochrome c oxidase subunit IV
CTL: control
DAPI: diamidino-2-phenylindole
DBZ: dibenzazepine
E13.5: embryonic day 13.5
E18.5: embryonic day 18.5
EGF: epidermal growth factor
FA: folic acid
FAO: fatty acid oxidation
FBS: fetal bovine serum
GEO: Gene Expression Omnibus
GFP: green fluorescent protein
ICN1: intracellular domain of NOTCH1
ICN2: intracellular domain of NOTCH2
ip: intraperitoneal
H&E: Hemotoxylin and Eosin
IRB: institutional review board
KEGG: Kyoto Encyclopedia of Genes and Genomes
LTL: lotus lectin
NICD: Notch intracellular domain
OCR: oxygen consumption rate
PAS: Periodic acid–Schiff
PNA: peanut agglutinin
PPARα: peroxisome proliferator-activated receptor α
P20: postnatal day 20
qRT-PCR: quantitative reverse transcriptase polymerase chain reaction
RBPJ: recombination signal binding protein for immunoglobulin kappa J region
RTEC: renal tubular epithelial cell
TGF-β1: transforming growth factor-β1
Tfam: mitochondrial transcription factor A
TIF: tubulointerstitial fibrosis
UUO: unilateral ureteral obstruction
