# Supplementary material for: Jagged1/Notch2 controls kidney fibrosis via Tfam-mediated metabolic reprogramming
Source: PLoS Biol. 2018 Sep 18;16(9):e2005233. doi: 10.1371/journal.pbio.2005233 (PMC6161902; doi:10.1371/journal.pbio.2005233)
Supplement: S1 Table — RBPJ, recombination signal binding protein for immunoglobulin kappa J region. (DOC) [file pbio.2005233.s002.doc]

| **S1 Table. RBPJ binding sites overlap with upregulated genes in *Pax8^rtTA^/TRE^ICNotch1^*** mice   | Chromosome | start | end | Associated Gene | RBPJ binding | | --- | --- | --- | --- | --- | | chr6 | 116594407 | 116595007 | *8430408G22Rik* | Inducible | | chr3 | 95491829 | 95492429 | *Adamtsl4* | Inducible | | chr5 | 97251538 | 97252138 | *Anxa3* | Inducible | | chr14 | 35260679 | 35261279 | *Bmpr1a* | Inducible | | chr1 | 178736644 | 178737244 | *Cep170* | Constant | | chr10 | 3156954 | 3157554 | *Cnksr3* | Inducible | | chr10 | 3227813 | 3228413 | *Cnksr3* | Inducible | | chr4 | 84443853 | 84444453 | *Cntln* | Inducible | | chr10 | 116874285 | 116874885 | *Cpsf6* | Constant | | chr8 | 37217734 | 37218334 | *D8Ertd82e* | Inducible | | chr12 | 105938345 | 105938945 | *Dicer1* | Inducible | | chr18 | 20192618 | 20193218 | *Dsc2* | Constant | | chr19 | 53631284 | 53631884 | *Dusp5* | Inducible | | chr19 | 53629606 | 53630206 | *Dusp5* | Inducible | | chr2 | 125101167 | 125101767 | *Dut* | Inducible | | chr2 | 125105413 | 125106013 | *Dut* | Inducible | | chr11 | 16708806 | 16709406 | *Egfr* | Inducible | | chr19 | 29703490 | 29704090 | *Ermp1* | Inducible | | chr13 | 96315537 | 96316137 | *F2rl1* | Inducible | | chr14 | 46149828 | 46150428 | *Fermt2* | Constant | | chr2 | 128254807 | 128255407 | *Gm14005* | Inducible | | chr2 | 128296053 | 128296653 | *Gm14005* | Inducible | | chr2 | 128370195 | 128370795 | *Gm14005* | Inducible | | chr12 | 112712876 | 112713476 | *Gm266* | Inducible | | chr1 | 74324372 | 74324972 | *Gpbar1* | Inducible | | chr16 | 30066154 | 30066754 | *Hes1* | Inducible | | chr16 | 30063927 | 30064527 | *Hes1* | Inducible | | chr16 | 30065039 | 30065639 | *Hes1* | Inducible | | chr3 | 8717326 | 8717926 | *Hey1* | Inducible | | chr4 | 122904856 | 122905456 | *Heyl* | Inducible | | chr4 | 122916604 | 122917204 | *Heyl* | Inducible | | chr4 | 122905468 | 122906068 | *Heyl* | Inducible | | chr2 | 136875009 | 136875609 | *Jag1* | Inducible | | chr1 | 138455324 | 138455924 | *Kif14* | Inducible | | chr13 | 5347082 | 5347682 | *Klf6* | Inducible | | chr17 | 73308955 | 73309555 | *Lbh* | Inducible | | chr13 | 104061069 | 104061669 | *Mast4* | Inducible | | chr14 | 120772066 | 120772666 | *Mbnl2* | Constant | | chr3 | 95471701 | 95472301 | *Mcl1* | Inducible | | chr16 | 13354044 | 13354644 | *Mkl2* | Inducible | | chr19 | 11663565 | 11664165 | *Ms4a6d* | Inducible | | chr1 | 137533926 | 137534526 | *Nav1* | Inducible | | chr1 | 43502048 | 43502648 | *Nck2* | Constant | | chr2 | 26349755 | 26350355 | *Notch1* | Inducible | | chr2 | 25033045 | 25033645 | *Nrarp* | Inducible | | chr2 | 25028029 | 25028629 | *Nrarp* | Inducible | | chr10 | 93105473 | 93106073 | *Ntn4* | Inducible | | chr16 | 92621330 | 92621930 | *Runx1* | Inducible | | chr3 | 93341215 | 93341815 | *S100a11* | Inducible | | chr13 | 93418835 | 93419435 | *Serinc5* | Inducible | | chr11 | 20208861 | 20209461 | *Slc1a4* | Inducible | | chr9 | 63550165 | 63550765 | *Smad3* | Inducible | | chr14 | 61330968 | 61331568 | *Spata13* | Inducible | | chr13 | 69724585 | 69725186 | *Srd5a1* | Inducible | | chr7 | 26496547 | 26497147 | *Tgfb1* | Inducible | | chr4 | 47306423 | 47307023 | *Tgfbr1* | Inducible | |
| --- | --- | --- | --- | --- | --- | --- | --- | --- | --- | --- | --- | --- | --- | --- | --- | --- | --- | --- | --- | --- | --- | --- | --- | --- | --- | --- | --- | --- | --- | --- | --- | --- | --- | --- | --- | --- | --- | --- | --- | --- | --- | --- | --- | --- | --- | --- | --- | --- | --- | --- | --- | --- | --- | --- | --- | --- | --- | --- | --- | --- | --- | --- | --- | --- | --- | --- | --- | --- | --- | --- | --- | --- | --- | --- | --- | --- | --- | --- | --- | --- | --- | --- | --- | --- | --- | --- | --- | --- | --- | --- | --- | --- | --- | --- | --- | --- | --- | --- | --- | --- | --- | --- | --- | --- | --- | --- | --- | --- | --- | --- | --- | --- | --- | --- | --- | --- | --- | --- | --- | --- | --- | --- | --- | --- | --- | --- | --- | --- | --- | --- | --- | --- | --- | --- | --- | --- | --- | --- | --- | --- | --- | --- | --- | --- | --- | --- | --- | --- | --- | --- | --- | --- | --- | --- | --- | --- | --- | --- | --- | --- | --- | --- | --- | --- | --- | --- | --- | --- | --- | --- | --- | --- | --- | --- | --- | --- | --- | --- | --- | --- | --- | --- | --- | --- | --- | --- | --- | --- | --- | --- | --- | --- | --- | --- | --- | --- | --- | --- | --- | --- | --- | --- | --- | --- | --- | --- | --- | --- | --- | --- | --- | --- | --- | --- | --- | --- | --- | --- | --- | --- | --- | --- | --- | --- | --- | --- | --- | --- | --- | --- | --- | --- | --- | --- | --- | --- | --- | --- | --- | --- | --- | --- | --- | --- | --- | --- | --- | --- | --- | --- | --- | --- | --- | --- | --- | --- | --- | --- | --- | --- | --- | --- | --- | --- | --- | --- | --- | --- | --- | --- | --- | --- | --- | --- | --- | --- | --- | --- | --- | --- | --- | --- | --- | --- | --- |
