# Supplementary material for: Jagged1/Notch2 controls kidney fibrosis via Tfam-mediated metabolic reprogramming
Source: PLoS Biol. 2018 Sep 18;16(9):e2005233. doi: 10.1371/journal.pbio.2005233 (PMC6161902; doi:10.1371/journal.pbio.2005233)
Supplement: S2 Table — RBPJ, recombination signal binding protein for immunoglobulin kappa J region. (DOC) [file pbio.2005233.s003.doc]

| **S2 Table. RBPJ bin****ding sites overlap with downregulated genes in *Pax8^rtTA^/TRE^ICNotch1^*** mice   | Chromosome | start | end | Associated Gene | RBPJ binding | | --- | --- | --- | --- | --- | | chr3 | 59836361 | 59836961 | *Aadac* | Inducible | | chr13 | 91903860 | 91904460 | *Acot12* | Inducible | | chr3 | 96993376 | 96993976 | *Acp6* | Inducible | | chr15 | 38655430 | 38656030 | *Atp6v1c1* | Inducible | | chr17 | 72481480 | 72482080 | *Clip4* | Inducible | | chr6 | 117329822 | 117330422 | *Cxcl12* | Inducible | | chr3 | 129389836 | 129390436 | *Egf* | Constant | | chr17 | 74153736 | 74154336 | *Ehd3* | Constant | | chr16 | 37615579 | 37616179 | *Hgd* | Inducible | | chr8 | 94325366 | 94325966 | *Irx3* | Constant | | chr10 | 106642147 | 106642747 | *Lin7a* | Inducible | | chr8 | 37249739 | 37250339 | *Lonrf1* | Inducible | | chrX | 16281500 | 16282100 | *Maoa* | Inducible | | chr17 | 87601850 | 87602450 | *Mcfd2* | Inducible | | chr1 | 94344895 | 94345495 | *Ndufa10* | Inducible | | chr6 | 101239295 | 101239895 | *Pdzrn3* | Inducible | | chr5 | 45519151 | 45519751 | *Qdpr* | Inducible | | chr1 | 158933051 | 158933651 | *Ralgps2* | Inducible | | chr2 | 27595697 | 27596297 | *Rxra* | Inducible | | chr8 | 6608829 | 6609429 | *Slc10a2* | Inducible | | chr11 | 113352046 | 113352646 | *Slc39a11* | Inducible | | chr10 | 70700475 | 70701075 | *Tfam* | Inducible | | chr15 | 36970843 | 36971443 | *Zfp706* | Inducible | |
| --- | --- | --- | --- | --- | --- | --- | --- | --- | --- | --- | --- | --- | --- | --- | --- | --- | --- | --- | --- | --- | --- | --- | --- | --- | --- | --- | --- | --- | --- | --- | --- | --- | --- | --- | --- | --- | --- | --- | --- | --- | --- | --- | --- | --- | --- | --- | --- | --- | --- | --- | --- | --- | --- | --- | --- | --- | --- | --- | --- | --- | --- | --- | --- | --- | --- | --- | --- | --- | --- | --- | --- | --- | --- | --- | --- | --- | --- | --- | --- | --- | --- | --- | --- | --- | --- | --- | --- | --- | --- | --- | --- | --- | --- | --- | --- | --- | --- | --- | --- | --- | --- | --- | --- | --- | --- | --- | --- | --- | --- | --- | --- | --- | --- | --- | --- | --- | --- | --- | --- | --- |
