# Supplementary material for: Jagged1/Notch2 controls kidney fibrosis via Tfam-mediated metabolic reprogramming
Source: PLoS Biol. 2018 Sep 18;16(9):e2005233. doi: 10.1371/journal.pbio.2005233 (PMC6161902; doi:10.1371/journal.pbio.2005233)
Supplement: S3 Table — (DOC) [file pbio.2005233.s004.doc]

| **S3 Table. qPCR primer sequences** | | |
| --- | --- | --- |
| Gene | Forward | Reverse |
| *m Acta2* | CTGACAGAGGCACCACTGAA | AGAGGCATAGAGGGACAGCA |
| *m c-Myc* | CAACGACAGCAGCTCGCCCA | CCGTGGGGAGGACTCGGAGG |
| *m Ccne1* | GCGGACACAGCTTCGGGTCT | GCGGACTGAAAGGTCGGAGCC |
| *m Col1a1* | TGCCTGGACCTCCTGGCGAGCGT | AGCAGGTCCGGGAGCACCACGTT |
| *m Col3a1* | ACAGCTGGTGAACCTGGAAG | ACCAGGAGATCCATCTCGAC |
| *m Col4a1* | GACAGCCAGGTTTGACAGGT | GGCAGCTCTCTCCTTTCTGA |
| *m Dll1* | CATGAACAACCTAGCCAATTGC | GCCCCAATGATGCTAACAGAA |
| *m Dll4* | GACCTGCGGCCAGAGACTT | GAGCCTTGGATGATGATTTGG |
| *m Fibronectin 1* | ACAAGGTTCGGGAAGAGGTT | CCGTGTAAGGGTCAAAGCAT |
| *m Hes1* | GCTACCGGACCAAGGAAGTTC | GAGCTAGACTGTTCTCAAAGTGAGTGA |
| *m HeyL* | TTCGGCATGAAGCGGCCCAG | CTCCAGCTTGGAGGAGCCCTGT |
| *m Jag1* | ACACAGGGATTGCCCACTTC | AGCCAAAGCCATAGTAGTGGTCAT |
| *m Jag2* | CGACTCACACTGCGCTTCA | TCGGATTCCAGAGCAGATAGC |
| *m Notch1* | ACAGTGCAACCCCCTGTATG | TCTAGGCCATCCCACTCACA |
| *m Notch2* | GGCATGTTGGGGAAAGCTAC | GGACACAAAGCAGGGGTGAG |
| *m Notch3* | GAGGACCTGGTTGAAGAATTGATC | TGCAGTTTTTCCCCTTTTATCC |
| *m Notch4* | GAGGACCTGGTTGAAGAATTGATC | TGCAGTTTTTCCCCTTTTATCC |
| *m Snai1* | TGGAAAGGCCTTCTCTAGGC | GGAGAATGGCTTCTCACCAG |
| *m Snai2* | GCTGGAACTTGTTTGCGCTTT | GTGGTTATTTCTTTGGCCAGCA |
| *m Tfam* | GAGCAGCTAACTCCAAGTCAG | GAGCCGAATCATCCTTTGCCT |
| *m Ubiquitin C* | GCCCAGTGTTACCACCAAGAAG | GCTCTTTTTAGATACTGTGGTGAGGAA |
| *m Vimentin* | GATCGATGTGGACGTTTCCAA | ATACTGCTGGCGCACATCAC |
| *r Fibronectin* | CATGGCTTTAGGCGAACCA | CATCTACATTCGGCAGGTATGG |
| *r Acta2* | GGCTCCATCCTGGCTTCTCTAT | GGCGTGACTTAGAAGCATTTGC |
| *r c-Myc* | TCTCGGCCGCTGCCAAACTG | TGGGCGAGCTGCTGTCGTTG |
| *r Ccna2* | ACCCCGAAAAAGTGGCGCCAG | GTCCTTAAGAGGCGCAACCCGC |
| *r Ccnb1* | AGGTGTGGGCAGCCAGAGGT | AGGCAGCAACTGCCACAGGC |
| *r Ccnd1* | AGGGGATTCAGGACGACTCT | GGGCAACCTTCCCAATAAAT |
| *r Ccnd2* | GAACCTGGCCGCAGTCACCC | GGCTGCTCCCACGCTTCCAG |
| *r Ccne1* | ATGTCCAAGTGGCCTACGTC | GCGAGGACACCATAAGGAAA |
| *r Cdh1* | CGTGGATGTGGTAGACGTGAA | TTCTCCGCAGGCACAAAAAT |
| *r Cdh2* | CAGAGAGTCGCCAAATGTCA | TTCACAAGTCTCGGCCTCTT |
| *r Col1a1* | AATGGTGCTCCTGGTATTGC | GGTTCACCACTGTTGCCTTT |
| *r Col4a1* | TGCGCAAGTTCAGCACCAT | GAGGCGAAGTTGCAGACGTT |
| *r Hes1* | TCACCTGAAAATGCTGCACACT | CGGTGTTAACGCCCTCACA |
| *r Hey1* | TGGTTCCGGTCGCTTCA | TTTCGGCAATTTGTGTGGTCTA |
| *r Ppara* | CCTTTTTGTGGCTGCTAT | TCCCTGCTCTCCTGTATG |
| *r Ppargc1a* | AGTGTGCTGCCCTGGTTGGTG | GGAGGGTCATCGTTTGTGGTC |
| *r Snai1* | TTAAGTCCAGGGACCTGTGG | TGAATACTGAGGGGCAGGAG |
| *r Snai2* | TCTGCAGACCCACTCTGATG | AGCAGCCAGACTCCTCATGT |
| *r Tfam* | GAAAGCACAAATCAAGAGGAG | CTGCTTTTCATCATGAGACAG |
| *r Ubiquitin C* | CACCAAGAAGGTCAAACAGGAA | AAGACACCTCCCCATCAAACC |
| *r Vimentin* | TTCCCTGAACCTGAGAGAAACTAAC | TGTTCTTTTGGAGTGGGTGTCA |
