# Supplementary figures and images for: Jagged1/Notch2 controls kidney fibrosis via Tfam-mediated metabolic reprogramming
Source: PLoS Biol. 2018 Sep 18;16(9):e2005233. doi: 10.1371/journal.pbio.2005233 (PMC6161902; doi:10.1371/journal.pbio.2005233)

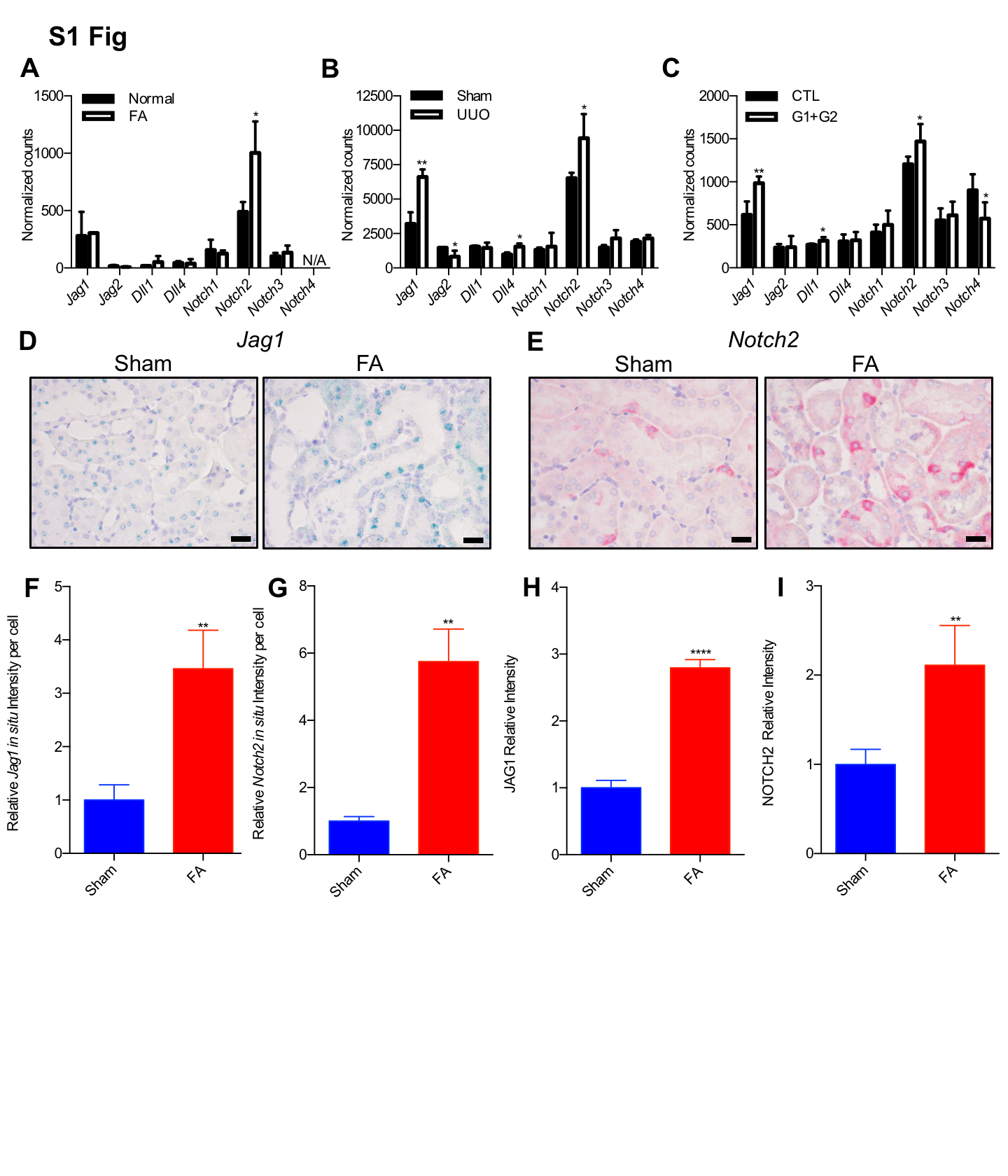

Supplement: S1 Fig — (A–C) Normalized counts of Notch ligands and receptors by RNA sequencing in whole-kidney lysates of FA-induced nephropathy group (n = 3 per group) (A), sham and UUO group (n = 4, 3) (B), and APOL1-G1/G2 mice (n = 6, 4) (C). Data are represented as the mean ± SD. * P < 0.05 and ** P < 0.01 by two-tailed Student t test. (D and E) Representative in situ hybridization images of Jag1 (D) and Notch2 (E) on sham and FA-treated mouse kidneys. Scale bar: 10 μm. (F and G) Relative Jag1 (F) and Notch2 (G) in situ hybridization intensity on sham and FA-treated mouse kidneys. ** P < 0.01 by two-tailed Student t test (n = 3 per group). (H and I) Relative JAG1 (H) and NOTCH2 (I) immunofluorescence intensity on sham or FA-treated mouse kidneys. ** P < 0.01 and **** P < 0.0001 by two-tailed Student t test (n = 4 per group). The underlying data of panels A, B, C, F, G, H, and I can be found in S1 Data. APOL1-G1/G2, apolipoprotein L1-G1 and G2 risk alleles; FA, folic acid; N/A, not available; UUO, unilateral ureteral obstruction. (TIFF) [file pbio.2005233.s005.tiff]

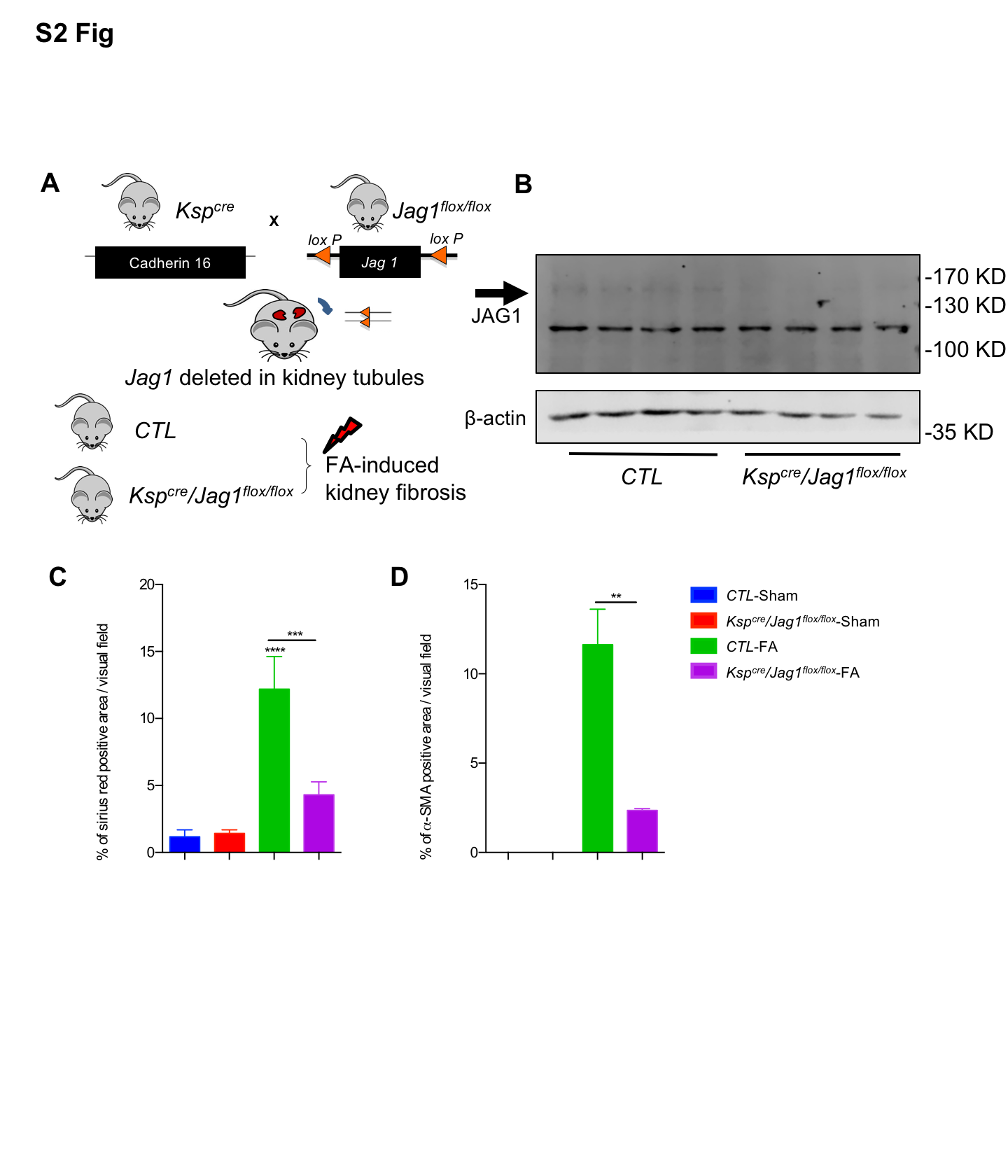

Supplement: S2 Fig — (A) Experimental scheme for generating the Kspcre/Jag1 flox/flox mice. Kidney injury was induced by FA injection. (B) Western blot analysis of JAG1 in whole kidney lysates of CTL and Kspcre/Jag1flox/flox mice. β-actin was used as a loading CTL. (C) Quantification of Sirius-Red–stained kidney sections from CTL and Kspcre /Jag1flox/flox mice with or without FA injection. Data are represented as mean ± SD. *** P < 0.001 and **** P < 0.0001 by one-way ANOVA with post hoc Tukey test (n = 3 per group). (D) Quantification of αSMA-stained kidney sections from CTL and Kspcre/Jag1flox/flox mice with or without FA injection. Data are represented as mean ± SD. ** P < 0.01 by two-tailed Student t test (n = 3 per group). The underlying data of panels C and D can be found in S1 Data. αSMA, alpha smooth muscle actin; CTL, control; FA, folic acid. (TIFF) [file pbio.2005233.s006.tiff]

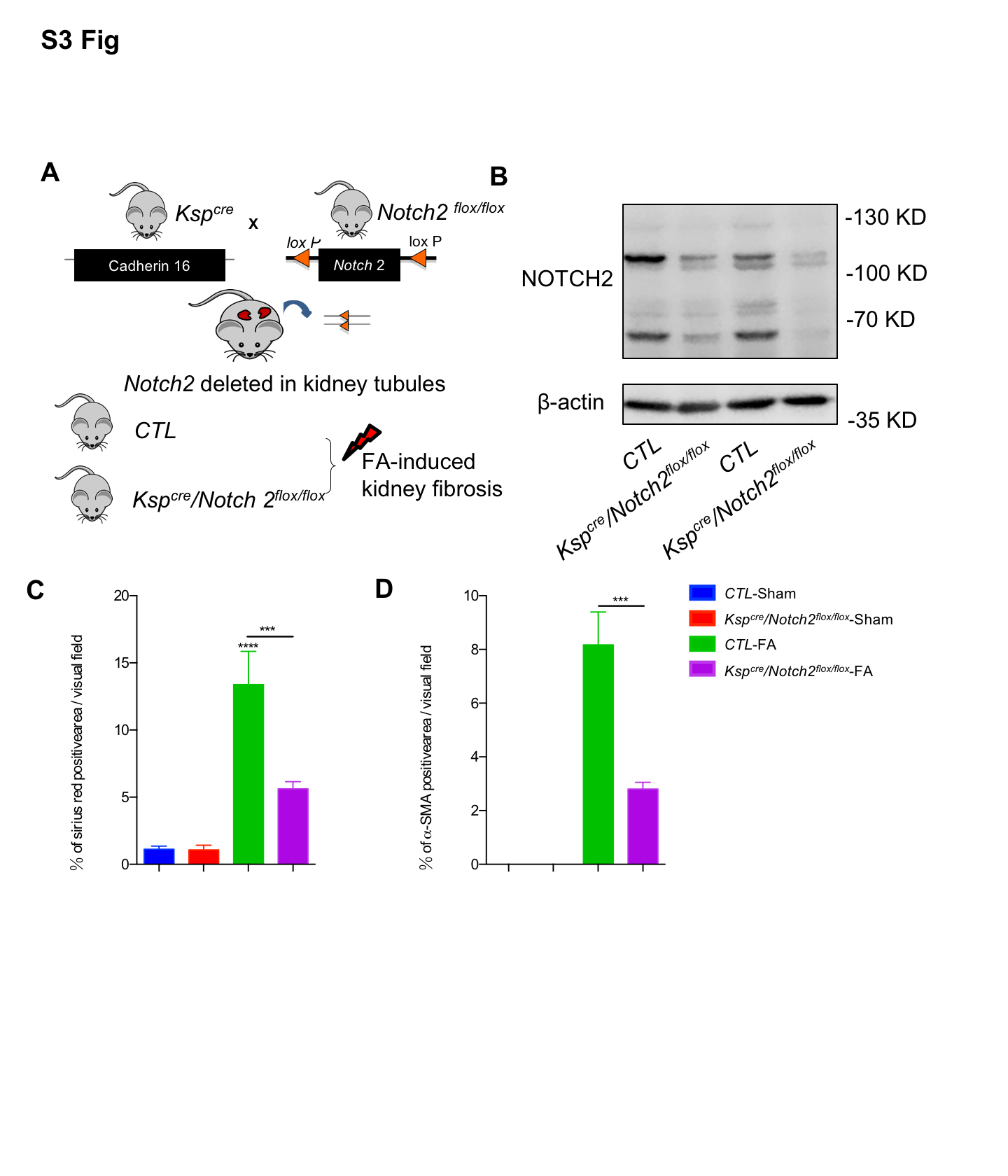

Supplement: S3 Fig — (A) Experimental scheme for generating the Kspcre/Notch2 flox/flox mice. Kidney injury was induced by FA injection. (B) Western blot analysis of NOTCH2 in whole kidney lysates of CTL and Kspcre/Notch2flox/flox mice. β-actin was used as a loading CTL. (C) Quantification of Sirius-Red–stained kidney sections from CTL and Kspcre /Notch2flox/flox mice with or without FA injection. Data are represented as mean ± SD. *** P < 0.001 and **** P < 0.0001 by one-way ANOVA with post hoc Tukey test (n = 3 per group). (D) Quantification of αSMA-stained kidney sections from CTL and Kspcre/Notch2flox/flox mice with or without FA injection. Data are represented as mean ± SD. *** P < 0.001 by two-tailed Student t test (n = 3 per group). The underlying data of panels C and D can be found in S1 Data. αSMA, alpha smooth muscle actin; CTL, control; FA, folic acid. (TIFF) [file pbio.2005233.s007.tiff]

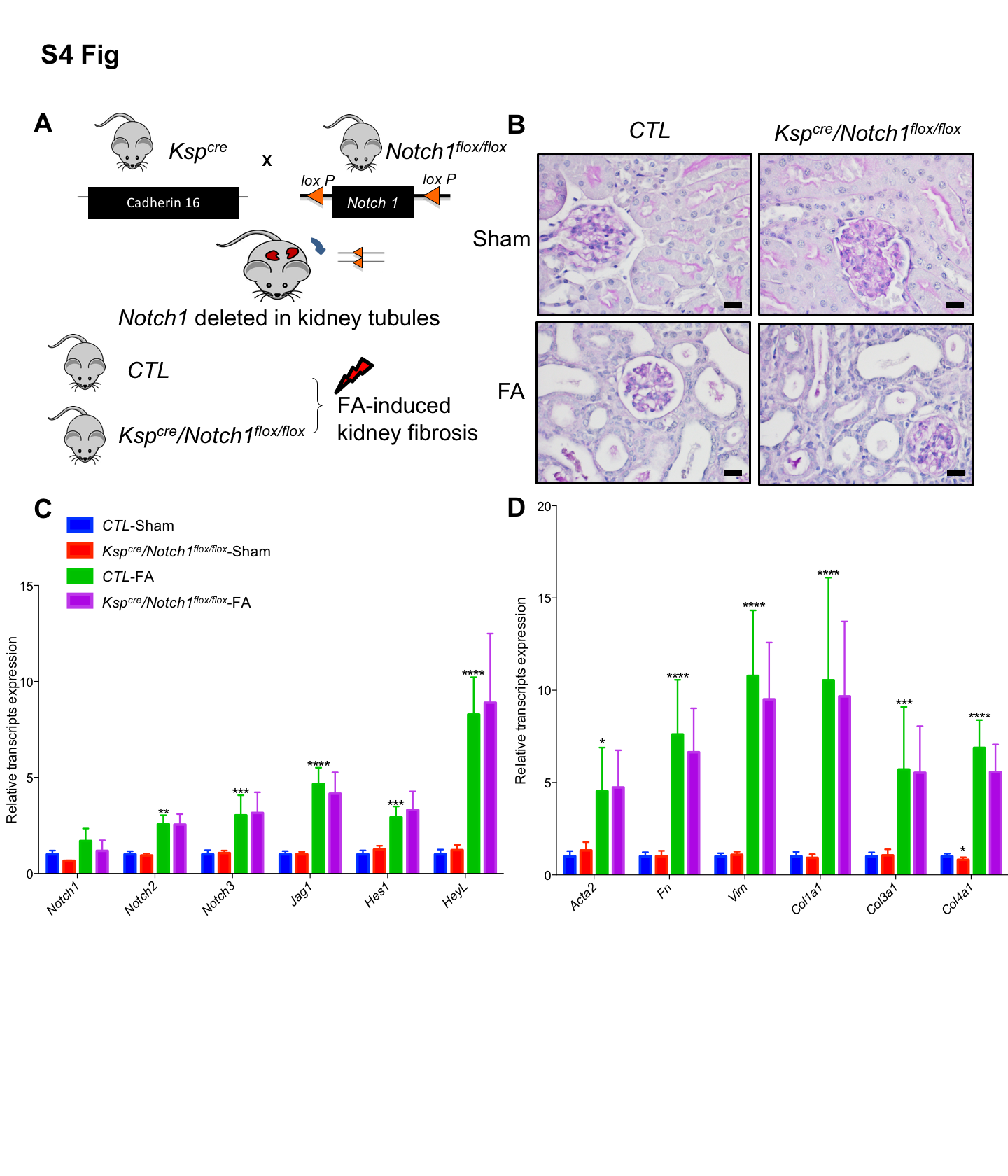

Supplement: S4 Fig — (A) Experimental scheme for generating the Kspcre/Notch1 flox/flox mice. Kidney injury was induced by FA injection. (B) Representative images of PAS-stained kidney sections from CTL and Kspcre /Notch1flox/flox mice with or without FA injection. Scale bar: 10 μm. (C and D) Relative mRNA amount of Notch signaling (C) and fibrosis markers (D) in CTL and Kspcre/Notch1flox/flox mice with or without FA injection. Data are represented as mean ± SD. * P < 0.05, ** P < 0.01, *** P < 0.001, and **** P < 0.0001 by two-way ANOVA with post hoc Tukey test (n = 7, 5, 6, 5). The underlying data of panels C and D can be found in S1 Data. CTL, control; FA, folic acid; PAS, Periodic acid–Schiff. (TIFF) [file pbio.2005233.s008.tiff]

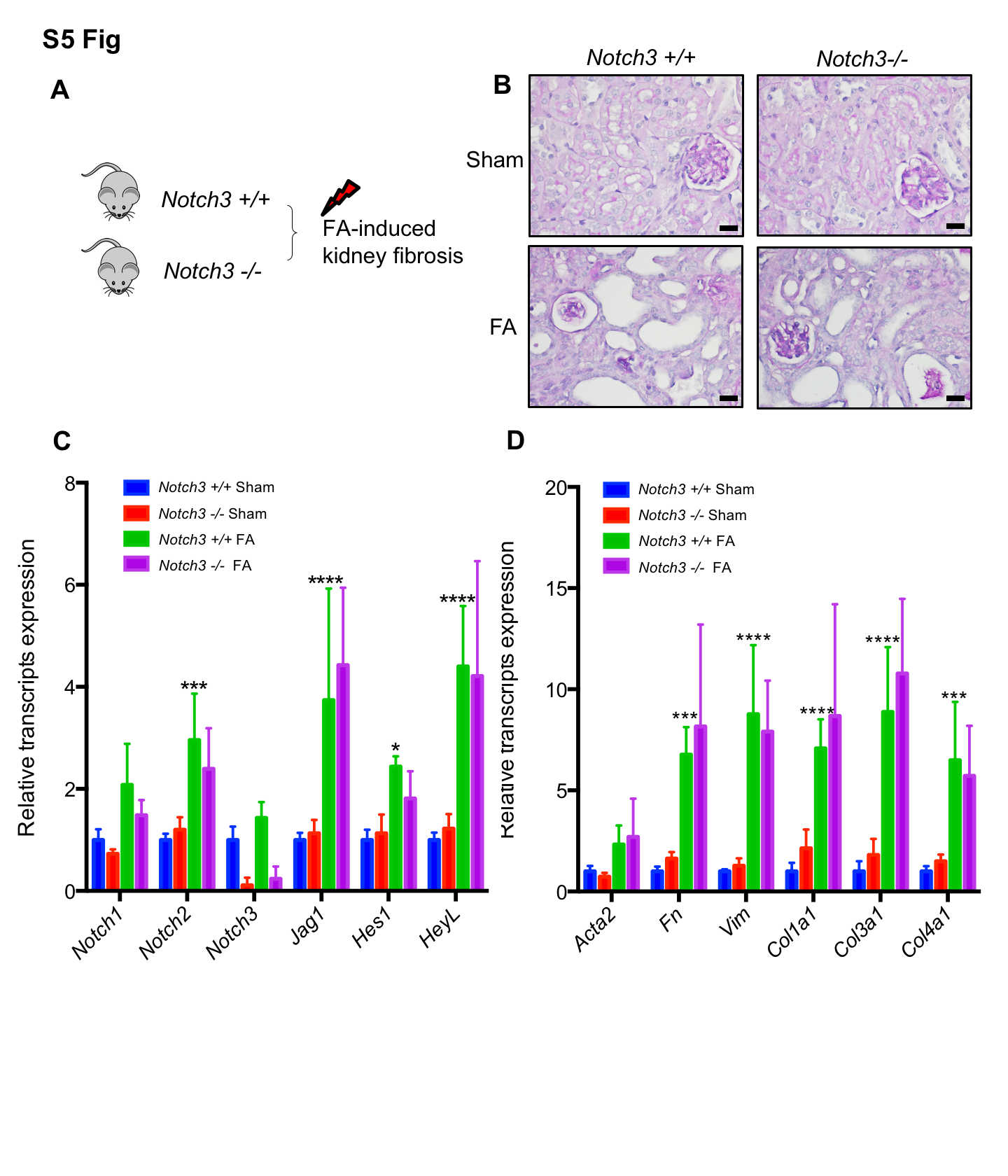

Supplement: S5 Fig — (A) Experimental scheme for generating the Notch3 knockout mice. Kidney injury was induced by FA injection. (B) Representative images of PAS-stained kidney sections from CTL and Notch3 knockout mice with or without FA injection. Scale bar: 10 μm. (C and D) Relative mRNA amount of Notch signaling (C) and fibrosis markers (D) in CTL and Notch3 knockout mice with or without FA injection. Data are represented as mean ± SD. * P < 0.05, *** P < 0.001, and **** P < 0.0001 by two-way ANOVA with post hoc Tukey test (n = 6, 5, 4, 4). The underlying data of panels C and D can be found in S1 Data. CTL, control; FA, folic acid; PAS, Periodic acid–Schiff. (TIFF) [file pbio.2005233.s009.tiff]

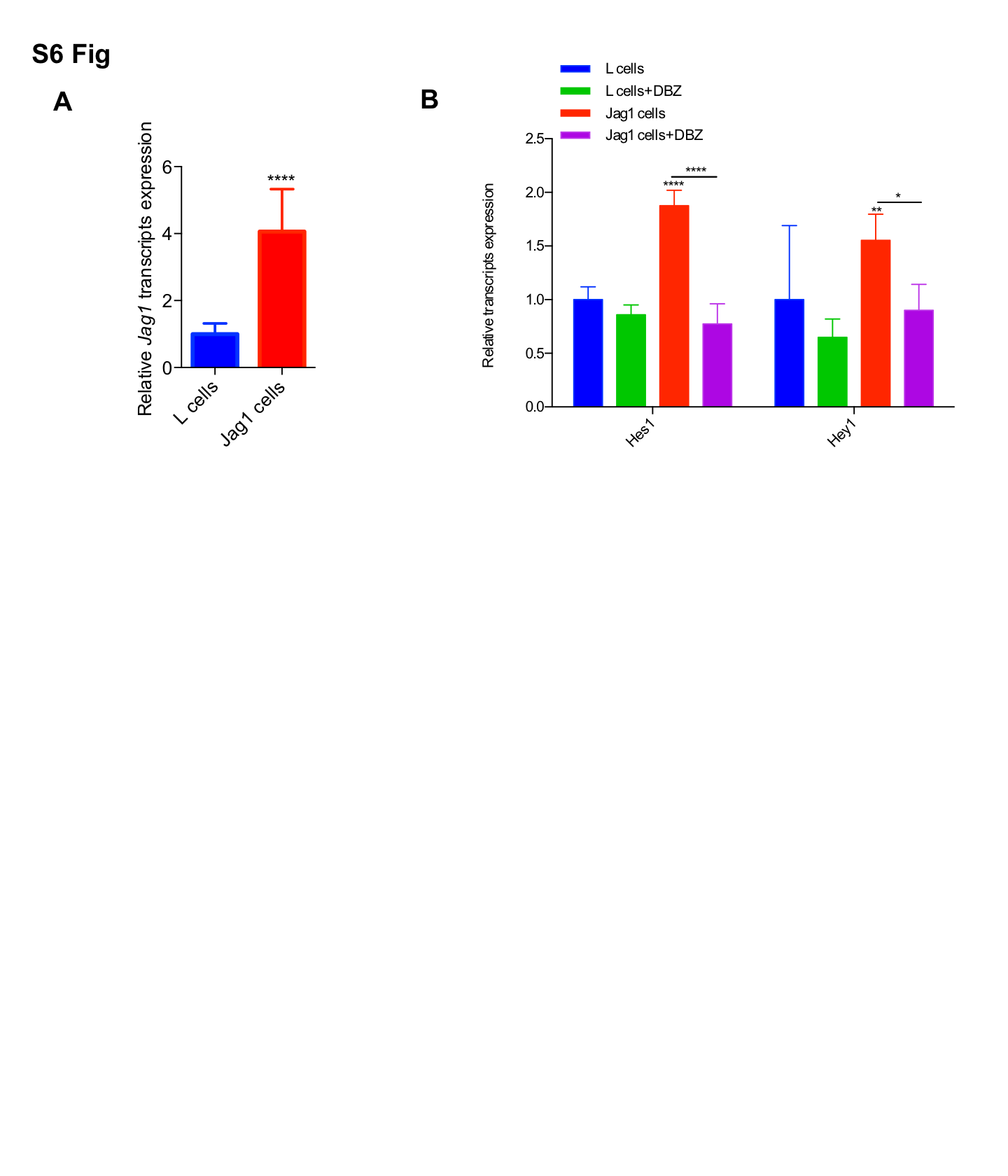

Supplement: S6 Fig — (A) Expression of mJag1 in JAG1-expressing L cells compared to CTL L cells. Data are represented as the mean ± SD. **** P < 0.0001 by two-tailed Student t test (n = 12 per group). (B) Relative mRNA amount of Hes1 and Hey1 in CTL and JAG1-expressing L cell with or without DBZ. Data are represented as mean ± SD. * P < 0.05, ** P < 0.01, and **** P < 0.0001 by two-way ANOVA with post hoc Tukey test (n = 8, 4, 8, 4). The underlying data of panels A and B can be found in S1 Data. CTL, control; DBZ, dibenzazepine. (TIFF) [file pbio.2005233.s010.tiff]

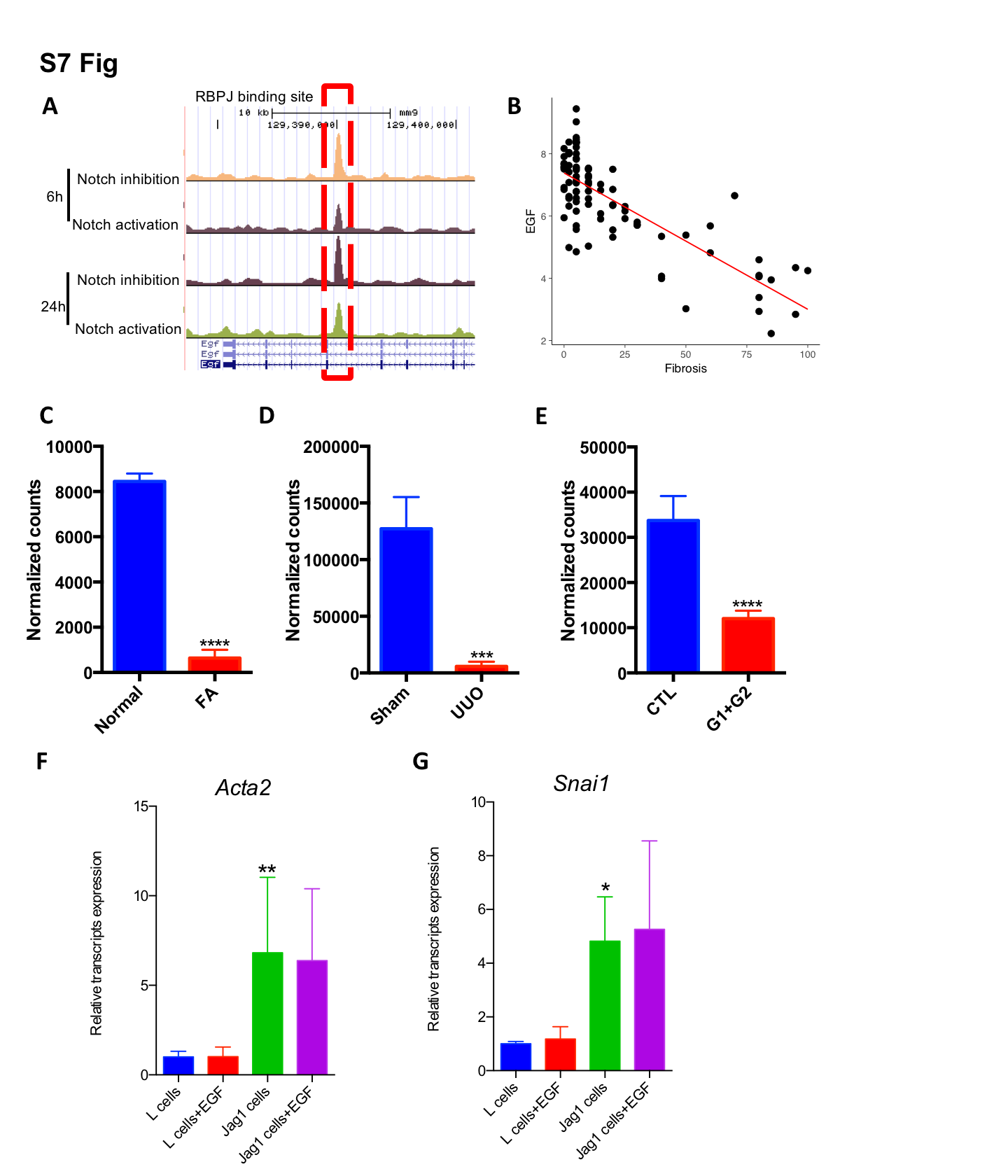

Supplement: S7 Fig — (A) The mouse Egf locus and RBPJ ChIP-Seq from 6 h and 24 h Notch activation and inhibition. (B) Correlation between interstitial fibrosis and EGF transcript level in 95 microdissected human kidney samples. (C–E) Normalized counts of Egf by RNA sequencing in whole-kidney lysates of FA-induced nephropathy group (n = 3 per group) (C), sham and UUO group (n = 4, 3) (D), and APOL1-G1/G2 mice (n = 6, 4) (E). Data are represented as the mean ± SD. *** P < 0.001 and **** P < 0.0001 by two-tailed Student t test. (F and G) Relative mRNA expression of Acta2 (F) and Snai1 (G) in JAG1 co-culture system when treated with EGF. Data are represented as mean ± SD. * P < 0.05 and ** P < 0.01 by one-way ANOVA with post hoc Tukey test (n = 8, 7, 4, 4). The underlying data of panels B, C, D, E, and F can be found in S1 Data. APOL1-G1/G2, apolipoprotein L1-G1 and G2 risk alleles; EGF, epidermal growth factor; FA, folic acid; RBPJ, recombination signal binding protein for immunoglobulin kappa J region; UUO, unilateral ureteral obstruction. (TIFF) [file pbio.2005233.s011.tiff]

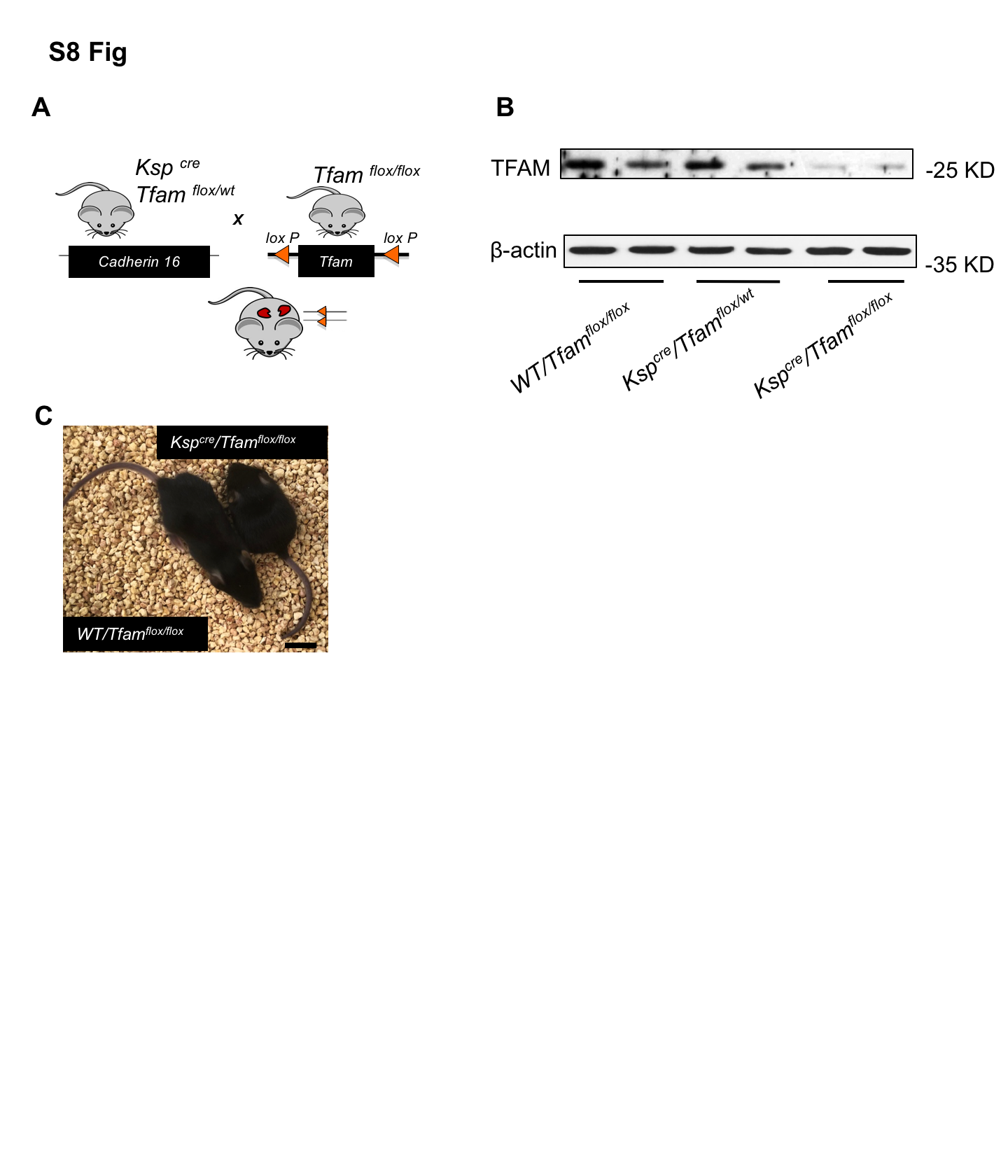

Supplement: S8 Fig — (A) Experimental scheme for generating the Kspcre/Tfamflox/flox mice. (B) Western blot analysis of TFAM in whole kidney lysates of WT/Tfamflox/flox, Kspcre/Tfamflox/wt and Kspcre/Tfamflox/flox mice. β-actin was used as a loading CTL. (C) Appearance of WT/Tfamflox/flox and Kspcre/Tfamflox/flox pups at postnatal day 15. Scale bar: 1 cm. CTL, control; Tfam, mitochondrial transcription factor A. (TIFF) [file pbio.2005233.s012.tiff]

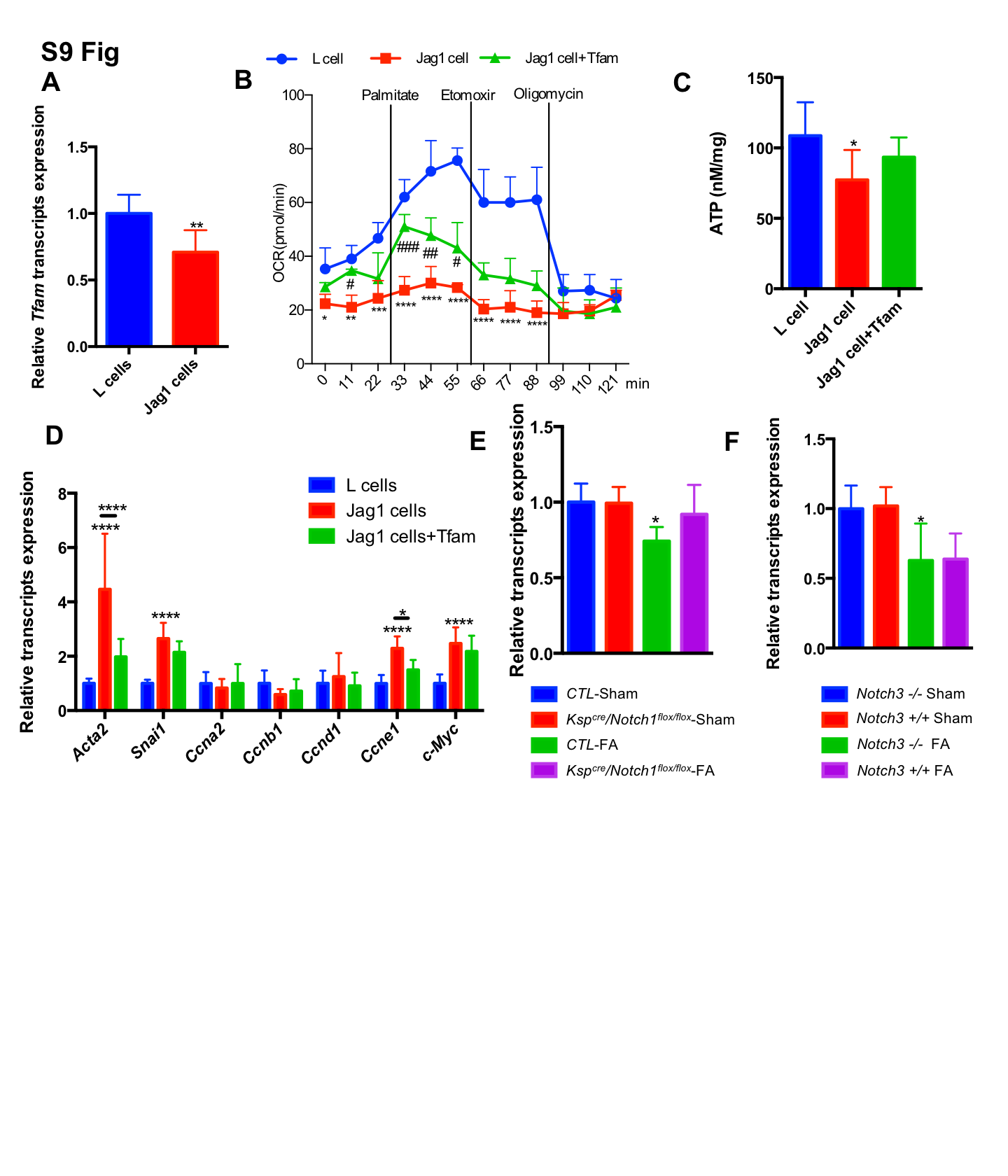

Supplement: S9 Fig — (A) Tfam expression level in JAG1 co-culture system. Data are represented as mean ± SD. ** P < 0.01 by two-tailed Student t test (n = 6 per group). (B) OCR in JAG1 co-culture system in the presence of GFP or TFAM plasmid. Where indicated, cells were incubated in palmitate (180 μM), etomoxir (40 μM), and oligomycin (1 μM). Data are represented as mean ± SD. *P < 0.05, ** P < 0.01, *** P < 0.001, and **** P < 0.0001 as compared to L cell group, # P < 0.05, ## P < 0.01, and ### P < 0.001 as compared to JAG1 cell group by two-way ANOVA with post hoc Tukey test (n = 3 per group). (C) ATP levels in JAG1 co-culture system in the presence of GFP or TFAM plasmid. Data are represented as mean ± SD. * P < 0.05 by one-way ANOVA with post hoc Tukey test (n = 8, 6, 6). (D) Relative mRNA expression of transcripts related to dedifferentiation and proliferation in JAG1 co-culture system in the presence of GFP or TFAM plasmid. Data are represented as mean ± SD. * P < 0.05 and **** P < 0.0001 by one-way ANOVA with post hoc Tukey test (n = 8 per group). (E) Relative mRNA amount of Tfam in CTL and Kspcre/Notch1flox/flox mice with or without FA injection. Data are represented as mean ± SD. * P < 0.05 by one-way ANOVA with post hoc Tukey test (n = 7, 5, 6, 5). (F) Relative mRNA amount of Tfam in CTL and Notch3 knockout mice with or without FA injection. Data are represented as mean ± SD. * P < 0.05 by one-way ANOVA with post hoc Tukey test (n = 6, 5, 4, 4). The underlying data of panels A–F can be found in S1 Data. CTL, control; FA, folic acid; GFP, green fluorescent protein; OCR, oxygen consumption rate; Tfam, mitochondrial transcription factor A. (TIFF) [file pbio.2005233.s013.tiff]

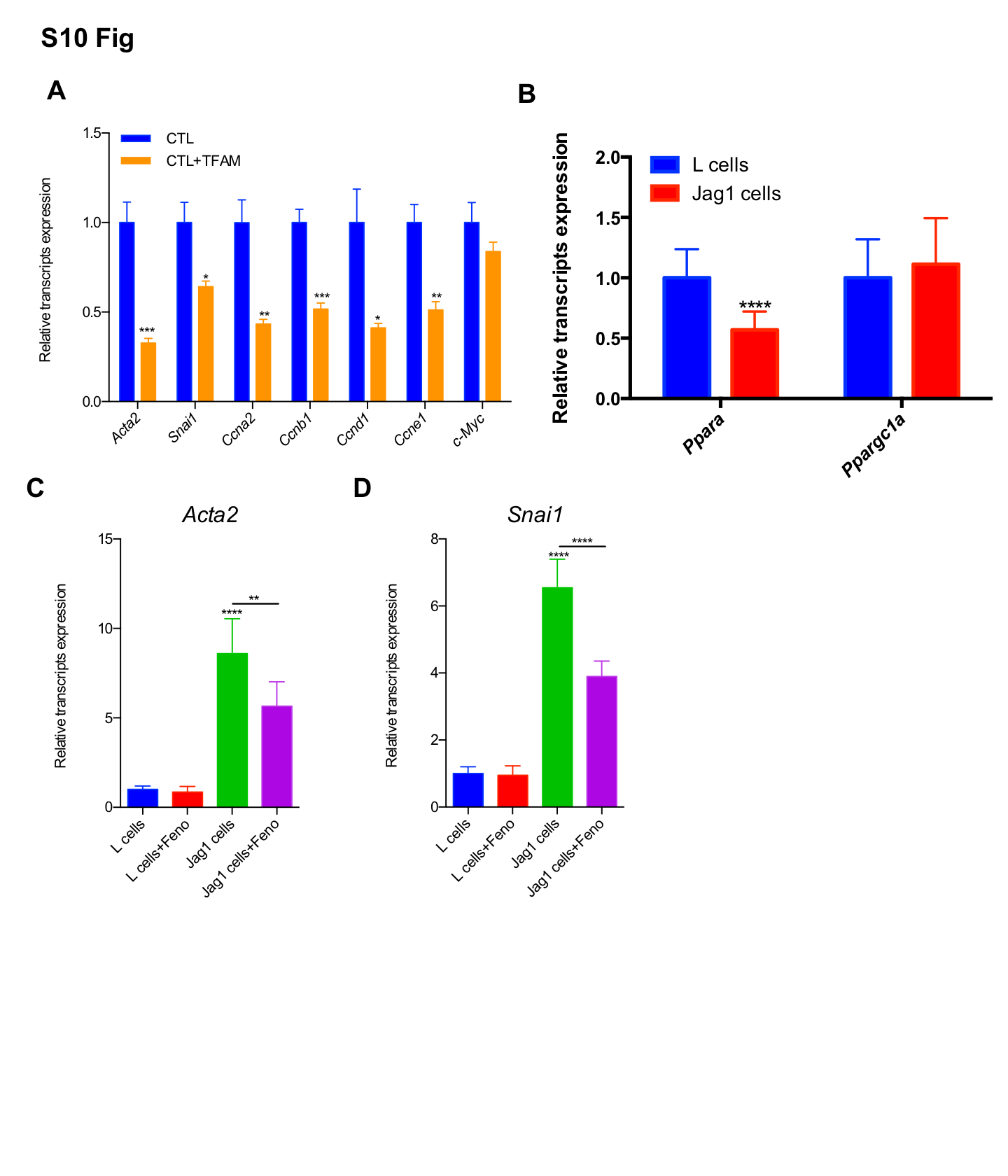

Supplement: S10 Fig — (A) Relative mRNA expression of transcripts related to fibrosis, dedifferentiation, and proliferation in NRK52E cells transfected with TFAM plasmids. Data are represented as mean ± SD. * P < 0.05, ** P < 0.01, and *** P < 0.001 by two-tailed Student t test (n = 6 per group). (B) Relative mRNA expression of Ppara and Ppargc1a in JAG1 co-culture system. Data are represented as mean ± SD. **** P < 0.0001 by two-tailed Student t test (n = 6 per group). (C and D) Relative mRNA expression of Acta2 (C) and Snai1 (D) in JAG1 co-culture system when treated with Feno. Data are represented as mean ± SD. ** P < 0.01 and **** P < 0.0001 by one-way ANOVA with post hoc Tukey test (n = 8, 7, 4, 4). The underlying data of panels A and B can be found in S1 Data. CTL, control; Feno, fenofibrate; PPARα, peroxisome proliferator-activated receptor α. (TIFF) [file pbio.2005233.s014.tiff]
